# IKKε and TBK1 in diffuse large B‐cell lymphoma: A possible mechanism of action of an IKKε/TBK1 inhibitor to repress NF‐κB and IL‐10 signalling

**DOI:** 10.1111/jcmm.15774

**Published:** 2020-08-28

**Authors:** Matthew Carr, Sami Mamand, Kathryn L. Chapman, Trevor Perrior, Simon D. Wagner

**Affiliations:** ^1^ Leicester Cancer Research Centre and Ernest and Helen Scott Haematological Research Unit University of Leicester Leicester UK; ^2^ Domainex Ltd., Chesterford Research Park Saffron Walden UK; ^3^ Present address: Polytechnic Research Center‐Erbil Polytechnic University Kurdistan Regional Government Erbil Iraq

**Keywords:** B‐cell, IKKε, lymphoma, TBK1

## Abstract

The IKK‐related kinases, IKKε and TBK1, have essential roles in innate immunity in part through modifying MYD88 signalling from the Toll‐like receptors to regulate NF‐κB signalling. We investigated the expression and function of IKKε and TBK1, in diffuse large B‐cell lymphoma (DLBCL). DLBCL cell lines and patient‐derived xenografts were used to determine their sensitivity to IKKε and TBK1 inhibitors. To understand the function of IKKε and TBK1 secreted factors were determined following administration of inhibitors. Gene expression microarrays were used to determine the transcriptional effects of inhibitors. Higher *TBK1* mRNA levels associated with poorer clinical outcome but IKKε and TBK1 were expressed in both germinal centre and non‐germinal centre types of DLBCL. Survival of cell lines Ly10, Ly03 and Pfeiffer, and of some primary human lymphoma cells, was suppressed by a small molecule IKKε/TBK1 inhibitor, DMX3433. DMX3433 reduced IL‐10 production from Ly10 and repressed NF‐κB mediated transcription. Inhibition of IKKε and TBK1 warrants further investigation as a potential therapeutic route to suppress NF‐κB signalling in lymphoma.

## 1 INTRODUCTION

Diffuse large B‐cell lymphomas (DLBCL) are highly heterogeneous but gene expression profiling has allowed classification into two major subgroups activated B‐cell‐like (ABC), germinal centre (GC) DLBCL and a third group (the ‘cell‐of‐origin’ [COO] classification).^1^, ^2^ More recently sequencing studies have produced a comprehensive cataloguing of mutations to define new subgroups of DLBCL^3^, ^4^, ^5^ of potential clinical significance. While some oncogenic drivers segregate into either ABC‐ or GC‐DLBCL others occur in both subgroups.^4^ More effective treatments are needed for those patients who respond poorly to the current standard combination chemotherapy regimens.^6^ Activation of NF‐κB signalling has an essential role in ABC‐DLBCL^7^, ^8^ and several routes to constitutive activation of NF‐κB signalling in this disease have been described including activating mutations of CARD11,^9^ MALT1^10^ and MYD88.^11^ Analysis of the most frequent MYD88 mutant (p.Leu265Pro) demonstrates that it promotes increased oligomerization propensity and constitutive NF‐κB signalling.^12^ Further investigation of circuitry downstream of NF‐κB shows that it drives cytokine (IL6 or IL10) production and JAK2/STAT3 activation in ABC‐DLBCL^11^, ^13^ (Figure S1). However, pre‐clinical studies suggest that a possible therapeutic route to NF‐κB inhibition (small molecule inhibition of IKKβ^14^) may be associated with compensatory IKKα signalling arguing against the feasibility of this therapeutic route.^15^

The standard‐of‐care treatment of DLBCL is combination immuno‐chemotherapy with rituximab, cyclophosphamide, adriamycin, vincristine and prednisolone (RCHOP), and this is curative in a small majority of patients although at the cost of morbidity and mortality due to neutropenic sepsis.^16^ Patients who are not cured clearly require different treatments and pre‐clinical studies have suggested that small molecule inhibitors of Bruton's tyrosine kinase (BTK)^17^ or phosphatidylinositol‐4,5‐bisphosphate 3‐kinase catalytic subunit delta (PI3Kδ)^18^ might be effective routes to inhibiting NF‐κB signalling. The effects of PI3Kδ, however, may be transient and reduced by a compensatory increase in PI3Kα,^19^ a result which seems to be reflected in a 30% response rate obtained in an early‐stage clinical trial of the small molecule PI3Kδ inhibitor, parsaclisib, in refractory or relapsed DLBCL.^20^ A phase III trial investigating the BTK inhibitor, ibrutinib, in combination with RCHOP in untreated DLBCL has been reported^21^ but the addition of ibrutinib did not improve event‐free survival. Other approaches to repressing NF‐κB signalling are, therefore, needed.

IKK‐related kinases IκB kinase ε (IKKε, also named IKKi or IKBKE)^22^, ^23^ and tank binding kinase 1 (TBK1) function in response to bacterial and viral signalling in normal immunity. TBK1 shows 64% homology to IKKε at the amino acid level.^24^ There are many uncertainties about the function of these kinases^25^ but it is clear that IKKε/TBK1 act downstream of Toll‐like receptors (TLRs) and MYD88, to phosphorylate interferon regulatory factors (IRF3 and IRF7) and induce type 1 interferon genes.^26^, ^27^ IKKε and TBK1 also regulate NF‐κB activity by directly phosphorylating RELA^24^, ^28^ controlling constitutive (but not cytokine‐induced) RELA phosphorylation at serine 536^29^ and promoting transactivation by p52 in the non‐canonical pathway.^30^ B‐cell specific roles have also been suggested and one study showed that TBK1 repressed the adapter NIK to prevent IgA class switching.^31^

The gene encoding IKKε, *IKBKE*, is amplified in 8 of 49 (16.3%) breast cancer cell lines and in over 30% of primary breast tumours^32^ and is essential to the survival of breast cancer cell lines through activation of NF‐κB suggesting that IKKε is a breast cancer oncogene.

Inhibition of IKKε and TBK1 is a potential route to repression of RELA activity but has not been previously considered as a potential therapeutic target in DLBCL. Here, we have employed small molecule inhibitors of IKKε/TBK1 to investigate the role of IKKε and TBK1 in DLBCL.

## 2 MATERIALS AND METHODS

### 2.1 Cell culture

Ly03 (RRID:CVCL_8800) and Ly10 (RRID:CVCL_8795) are representative of ABC‐DLBCL and Ly19 (RRID:CVCL_1878), SUDHL4 (RRID:CVCL_0539) and SUDHL6 (RRID:CVCL_2206) of GC‐DLBCL. Pfeiffer (RRID:CVCL_3326) and Toledo (RRID:CVCL_3611) are classified as Type III.^33^ Cell lines were obtained from ATCC and their identity was confirmed by STR testing in 2016. Ly03, Ly19, SUDHL4, SUDHL6, Toledo and Pfeiffer cell lines were cultured in RPMI 1640 medium containing L‐glutamine (Invitrogen, Thermo Fisher Scientific), and supplemented with 10% foetal calf serum (FCS) (Lonza). Ly10 cells were grown in IMDM containing L‐glutamine and 25 mmol/L HEPES (Invitrogen), and supplemented with 10% FCS (Lonza). Structures and details of medicinal chemistry of IKKε/TBK1 inhibitors can be found in US patent 8962609 B2 "Pyrimidine compounds as inhibitors of protein kinases IKK epsilon and/or TBK1, processes for their preparation and pharmaceutical compositions containing them". For viability assays, equal numbers of cells were seeded in technical duplicate in fresh media with vehicle control (DMSO only) wells in a 96‐well plate. Cells were then incubated for 48 hours in standard tissue culture conditions. Cell viability was assessed using CellTiter‐Glo^®^ (Promega) and relative cell survival was analysed in GraphPad Prism 7.0 and a sigmoidal, 4PL curve was fitted to generate EC50 values.

### 2.2 Western blotting

Cell lysates were produced and Western blotting was carried out as described previously.^34^ Primary antibodies used were all from Cell Signalling Technology: phosphorylated‐AKT (S473) (#4060), AKT (#4691), phosphorylated RELA (S536) (#3033), RELA (#8242), pSTAT3 (s727) (#9134), phosphorylated‐STAT3 (Y705) (#9145), STAT3 (#4904), IKKε (#2904), TBK1 (#3013), β‐actin (#8457), GAPDH (#2118). Membranes were incubated with HRP‐coupled anti‐rabbit secondary antibody (Cell Signaling Technology), in 5% milk/BSA in TBS‐0.05% Tween‐20 for 1 hour at room temperature and visualization was with ECL (BioRad) and exposure to X‐ray film.

### 2.3 Luminex assay

Using information from the literature, a Luminex panel was designed to account for the major growth factors and chemokines produced by B cells. Initial experiments comparing Ly03 and Ly10 showed that Ly10 produced the greatest amounts of growth factor in baseline conditions. For further experiments, cell culture supernatants were harvested from Ly10 cells, centrifuged at 310 *g* for 5 minutes, to remove debris and stored at −80°C prior to analysis. Tumour necrosis factor α (TNFα), interferon β (IFNβ), lymphotoxin α (LTα), CXCL6, CXCL13, CCL3, CCL4, CCL17, CCL22, IL2, IL4, IL6, IL9, IL10, IL12 and IL13 were analysed by magnetic Luminex assay (R&D Systems). Assay plates were read in a Luminex MAGPIX system with xPONENT software (Luminex).

### 2.4 Taqman assay

Total mRNA was extracted from harvested cells using a RNeasy Mini Kit (Qiagen). Reverse transcription was carried out with the SensiFAST™ cDNA synthesis kit using the manufacturer's protocol (Bioline). Reactions were then carried out using Taqman primers for IL10 (Hs00961622_m1) and HPRT (Hs02800695) (Applied Biosystems).

### 2.5 Immunohistochemistry and Immunofluorescence

A human DLBCL tissue microarray was used consisting of 72 cases of DLBCL (catalog number LY1001c; US Biomax Inc) of which 7 cases could not be used. The GC/non‐GC status can be found at https://www.biomax.us/tissue‐arrays/Lymphoma/LY1001c.

Multiplexed immunohistochemical staining was performed with the Opal IHC Kit (PerkinElmer). Antibodies were diluted as follows: anti‐IKKε (1:100) and anti‐TBK1 (1:100). Formalin‐fixed and paraffin‐embedded (FFPE)‐TMA sections were microwaved in Tris‐EDTA (pH 9.0) at 700 W for 20 minutes following incubation with protein block (X0909; DAKO) for 10 minutes. Sections were incubated with anti‐IKKε and anti‐TBK1 antibodies for 30 minutes at room temperature. Secondary Opal™ Polymer HRP Ms + Rb (ARH1001EA) was incubated for 30 minutes at room temperature, followed by washing steps. The slides were then incubated for 10 minutes at room temperature in the dark with Opal 520 (diluted 1:200) for anti‐IKKε and Opal 570 (diluted 1:200) for anti‐TBK1. The sections were counterstained with DAPI for 5 minutes, then mounted with anti‐fade mountant (P36930; Dako). Negative control rabbit (ab172730; Abcam) was used in each staining run. Images were obtained using Vectra Polaris multi‐colour fluorescence scanner (Akoya Biosciences), and the quantitative analysis was performed by the use of inForm software (Akoya Biosciences) (Table S1).

### 2.6 Patient‐derived xenograft models

All animal studies were conducted at Crown Bioscience HuPrime SPF animal facility (CrownBio) under sterile conditions and were in strict accordance with the Guide for the Care and Use of Laboratory Animals of the National Institutes of Health. Protocols of all studies were approved by the Committee on the Ethics of Animal Experiments of Crown Bioscience, Inc (Crown Bioscience IACUC Committee). The patient‐derived xenograft models were obtained from Crown Bioscience. Tumour growth was monitored twice weekly using a caliper and all efforts were made to minimize suffering.^35^ Animals were euthanized by CO_2_ inhalation. Characteristics of PDX models used (PDX0257, PDX2345, PDX2214 and PDX2318) are presented (Table S2).

Ex vivo 2D cultures were set up at a cell concentration of 1 × 10^5^/mL in a 96‐well plate. Viability following the addition of drug was measured at 24 hours using CellTiter‐Glo^®^ (Promega).

To generate cell pellets, 2 × 10^6^ cells were seeded in 1.9 mL of X‐vivo 15 basal growth medium per well of a 6‐well plate. Cells were then incubated overnight, followed by incubation for 24 hours with drug or vehicle control (DMSO). Post incubation, cell supernatant was removed, and the cells were harvested and centrifuged. Cell pellets were then stored at −80°C prior to shipping on dry ice.

### 2.7 Gene expression microarray analysis

Total RNA was purified from PDX model cell pellets. RNA isolation was carried out by means of Trizol/chloroform phase separation followed by PureLink^®^ RNA Mini Kit (ThermoFisher Scientific) procedure. RNA quality was checked on a Bioanalyzer 2100 (Agilent). All RNA samples had a RNA Integration Number (RIN) > 7. A total of 100 ng of total RNA were reverse transcribed, converted into complementary RNA (cRNA) and labelled with Cy3 using the LowInput QuickAmp Labeling Kit One‐Color according to manufacturer's protocol (Agilent). Labelled cRNA was then hybridized over night at 65°C onto the SurePrint G3 Human Gene Expression v3 8 × 60 K Microarray and scanned on an Agilent DNA microarray C‐scanner. Extraction and quality check of the raw data were performed using the Agilent Feature extraction software version 10.5.1.1.

Quantile normalization of data was performed using Partek Genomic suite (Partek Inc). Normalized data were then imported into Multiple Experiment Viewer (MeV),^36^ and a two‐class unpaired significance analysis for microarrays (SAM) analysis was performed. Hierarchical clustering (distance metric selection by Pearson correlation, linkage by average linkage clustering) was carried out for significant genes (FDR q‐value of <0.25 and a nominal *P* value of <.05). The data that support the findings of this study are openly available in Gene Expression Omnibus (GEO) at https://www.ncbi.nlm.nih.gov/geo/, reference number GSE121159.

### 2.8 Gene set enrichment analysis

Gene expression values from untreated and treated PDX models were analysed with the desktop GSEA software (Broad institute). The PDX expression set was interrogated for enrichment with hallmark gene sets in the Broad molecular signature database. Enrichment with pathways was determined using thresholds of a FDR q‐value of <0.25, a normalized enrichment score <−1 or >1 and a nominal *P*‐value of <.05.

### 2.9 Kaplan‐Meier survival analysis

Survival data were generated using SurvExpress^37^ and data from a gene expression data set profiling 420 DLBCL patients treated with either CHOP or R‐CHOP regimens (GSE10846, Lenz et al 2008). Data were then subjected to Kaplan‐Meier survival analysis using GraphPad Prism 6.0.

## 3 RESULTS

### 3.1 IKBKE and TBK1 mRNA expression and prognosis in DLBCL

**FIGURE 1:**
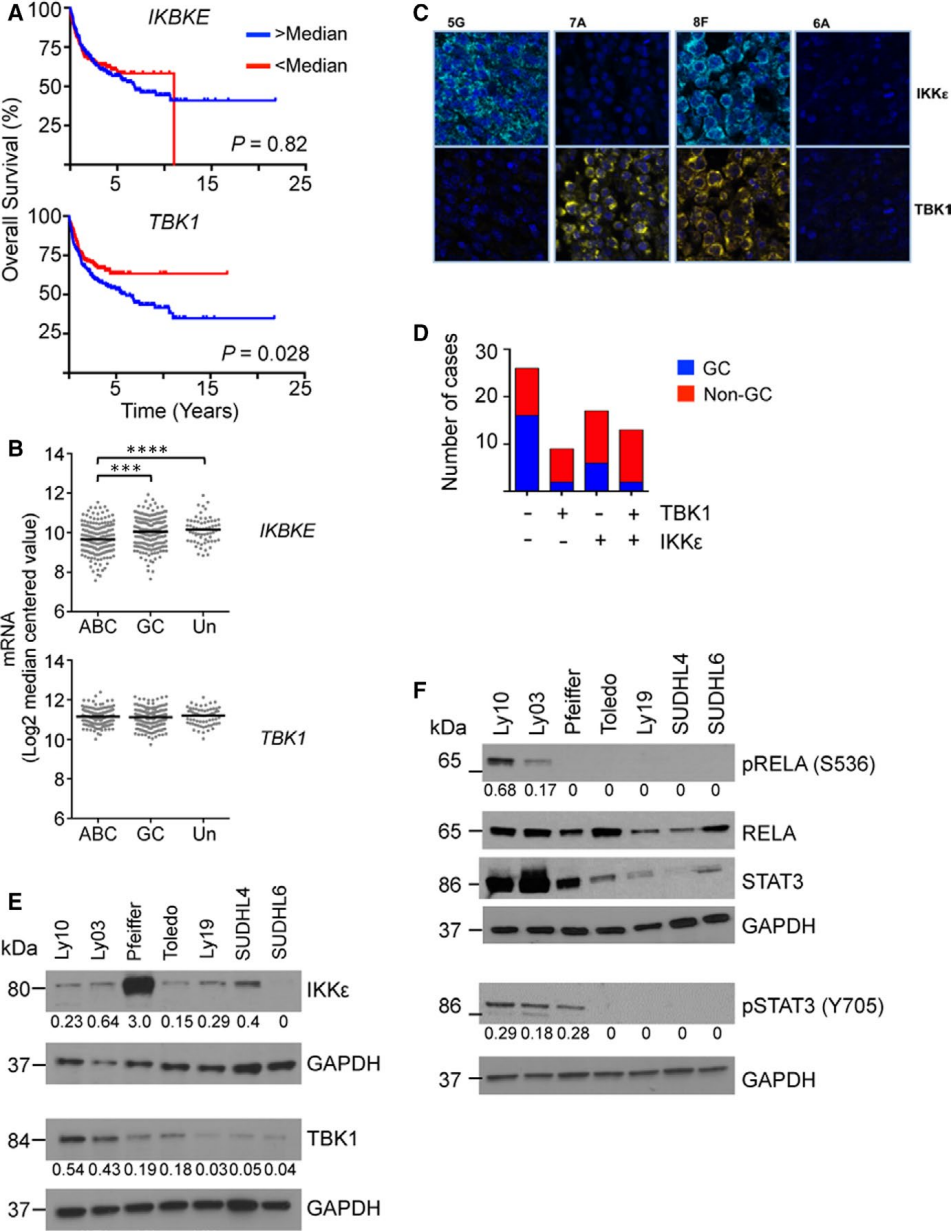
Expression of IKKε and TBK1 in DLBCL. A, Kaplan‐Meier plots showing overall survival (%) by *IKBKE* (upper panel) and *TBK1* (lower panel) levels. For each plot, the blue line represents patients with mRNA levels greater than the median and the red line those patients with levels less than the median. Data on expression and survival is from GSE10846^38^ using *TBK1* probeset 218520_at and *IKBKE* probeset 204549_at. Higher TBK1 levels are associated with poor clinical outcome (log‐rank test, *P* = .042), which is not observed for IKBKE levels (*P* = .88). B, Expression of *IKBKE* and *TBK1* mRNA in ABC, GC and unclassified (Un) subgroups of DLBCL. Data taken from,^38^ GEO dataset GSE10846. Median is indicated by the horizontal bar. For IKBKE median levels of GC cases (Mann‐Whitney test: *P* = .0002) and unclassified (*P* = <.0001) are significantly higher than levels in ABC cases. C, Immunofluorescence images from representative cases on the DLBCL TMA. The antibody used is indicated to the right either anti‐TBK1 (yellow) or anti‐IKKε (green). Both have also been stained with DAPI to define cell nuclei (blue). Representative examples are shown with their position on the microarray indicated by the letter and number above the images. IKKε^hi^/TBK1^lo^ (5G), IKKε^lo^/TBK1^hi^ (7A), IKKε^hi^/TBK1^hi^ (8F) and IKKε^lo^/TBK1^lo^ (6A). D, Histograms showing numbers of DLBCL cases (defined by expression of IKKε and TBK1) of GC (blue) or non‐GC (red) type in the tissue microarray of DLBCL. E, Western blots showing IKKε and TBK1 expression in a panel of DLBCL cell lines. GAPDH is a loading control. Molecular weight (kDa) is indicated to the left. The numbers below the IKKε and TBK1 autorads are ratios of IKKε:GAPDH and TBK1:GAPDH respectively. F, Expression of the NF‐κB component RELA and phosphorylated RELA together with STAT3 and phosphorylated STAT3 (Y705) in a panel of DLBCL cell lines. GAPDH is a loading control. The numbers below the pRELA and pSTAT3 autorads are ratios of pRELA/GAPDH:RELA/GAPDH and pSTAT3/GAPDH:STAT3/GAPDH respectively. Uncropped autorads are in the Appendix

Analysis of a publicly available gene expression data set (GSE10846^38^) showed that, across the entire cohort of patients with DLBCL, those expressing *TBK1* at levels greater than the median had significantly worse overall survival (log‐rank test, *P* = .028) (Figure 1A). IKBKE levels did not associate with survival (Figure S2). *TBK1* mRNA levels did not associate with differences in patient characteristics in respect of age, clinical stage and performance score (Table S3). Inspection of mRNA levels showed no significant *TBK1* differences across patient groups (Figure 1B) while *IKBKE* levels were higher in unclassified (Mann‐Whitney test: *P* = <.0001) and GC‐DLBCL (*P* = .0002) than in ABC‐DLBCL. Overall despite mRNA levels of *IKBKE* associating with specific subgroups of DLBCL, this correlation does not appear to influence patient survival. However, independent of COO subgroups higher *TBK1* mRNA levels associated with poorer clinical outcomes.

### 3.2 IKKε and TBK1 expression in primary DLBCL

Next, using a tissue microarrays (n = 65) (Figure 1C, D), we investigated IKKε and TBK1 protein expression in human DLBCL. 26/65 (40%) of cases showed expression of neither IKKε nor TBK1. TBK1 alone was detected in 9/65 (14%), while IKKε alone was found in 17/65 (26%). Cases expressing both IKKε and TBK1 were detected in 13/65 (20%). The majority of non‐GC‐DLBCL expressed either IKKε or TBK1 or both proteins (29/39 (74%)) but protein expression was not detectable in 16/26 (61%) of GC‐DLBCL. The difference in IKKε and TBK1 expression between GC and non‐GC cases was significant (chi‐square test; *P* = .022) (Table S4). IKKε and TBK1 are, therefore, found in both GC‐ and non‐GC cases with a greater proportion of non‐GC cases than GC cases showing expression.

Overall higher *TBK1* mRNA levels associate with poor clinical outcome but there is no difference in *TBK1* levels between GC‐ and non–GC‐DLBCL. However, TBK1 protein levels varied from undetectable to strongly expressed on the TMA. This suggests post‐transcriptional control contributes to the regulation of TBK1 protein levels.

### 3.3 IKKε and TBK1 expression in DLBCL cell lines and sensitivity to small molecule IKKε/TBK1 inhibitors

We next determined IKKε and TBK1 expression in a panel of seven DLBCL cell lines (Figure 1E). Western blots showed the lines Ly10, Ly03, Pfeiffer and Toledo to have the highest expression of TBK1. IKKε expression was greatest in Pfeiffer (a type 3 DLBCL cell line^33^) but this protein was detectable in all cell lines apart from the GC‐DLBCL line, SUDHL6. Therefore, in this panel of cell lines, and in‐line with the TMA results, IKKε and TBK1 expression did not clearly associate with COO subgroup. One of the principal relevant targets of IKKε/TBK1 in the NF‐κB pathway is RELA. All cell lines showed expression of RELA but only Ly10 and Ly03 showed detectable phosphorylated RELA at serine 536 (Figure 1F). Ly10 and Ly03 are known to bear the MYD88 change (p.Leu265Pro) which drives constitutive NF‐κB activity,^12^ while the other cell lines in the panel do not show the mutation.

**FIGURE 2:**
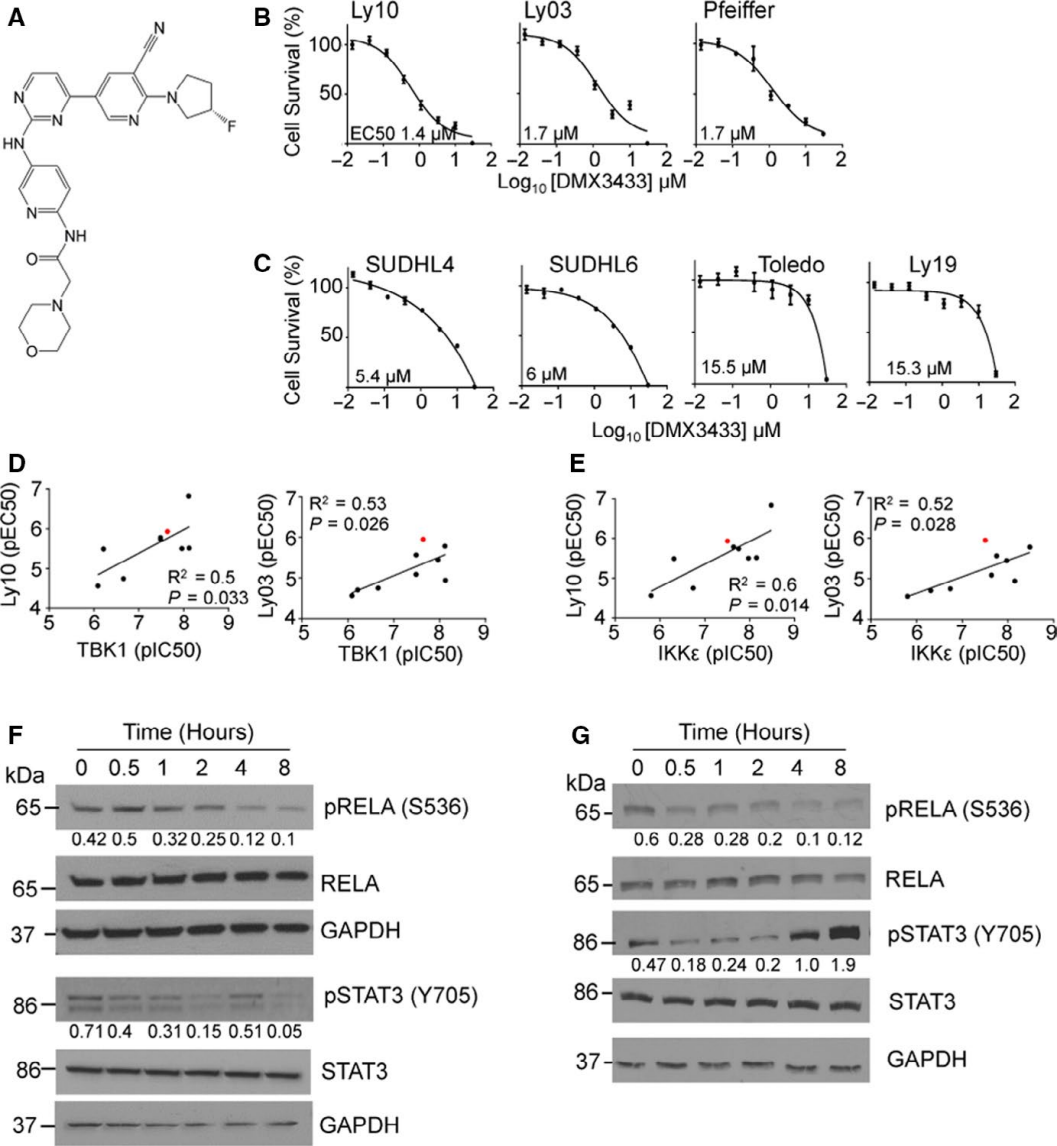
Sensitivity of DLBCL lines to a IKKε/TBK1 inhibitor. A, Chemical structure of DMX3433. B and C, Dose‐response curves showing mean ± SEM cell survival (%) determined by ATP luminescence in response to treatment with DMX3433 for 48 h. IC50 (µmol/L) are shown at lower right. B, Dose‐response curves for Ly03, Ly10 and Pfeiffer (n = 3). C, Dose‐response curves for SUDHL4 (n = 1), SUDHL6 (n = 3), Toledo and Ly19 (n = 3). D and E, Nine IKKε/TBK1 inhibitors were assessed for effects on survival (determined by ATP luminescence) of the Ly10 and Ly03 cell lines. DMX3433 is indicated by the red dot. Inhibition of cell line growth (pEC50) (y‐axis) is plotted against the concentration required for 50% inhibition (pIC50) of the purified enzyme (x‐axis) (D) TBK1 enzyme activity or (E) IKKε activity. F and G, Time course (0, 0.5, 1, 2, 4 and 8 h) showing changes in, phosphorylated RELA (serine 536) and total RELA, phosphorylated STAT3 (tyrosine 705), total STAT3in response to DMX3433 (2 µmol/L) in (F) Ly10 and (G) Ly03. The numbers below the pRELA and pSTAT3 autorads are ratios of pRELA/GAPDH:RELA/GAPDH and pSTAT3/GAPDH:STAT3/GAPDH respectively. GAPDH is a loading control. Uncropped autorads are in the Appendix.

In order to determine the effects of IKKε/TBK1 inhibition on DLBCL cell line growth, we used DMX3433 (Figure 2A), an IKKε/TBK1 inhibitor with pIC50 for the purified enzymes of 7.8 for IKKε and 7.9 for TBK1, which was available in sufficient quantities for all cellular and in vivo studies. We demonstrated inhibition of DLBCL cell line viability; Ly10, Ly03 and Pfeiffer being the most sensitive (EC50 of 1.4, 1.7 and 1.7 µmol/L, respectively) (Figure 2B), SUDHL4 (EC50 5.4 µmol/L) and SUDHL6 (EC50 6 µmol/L) being slightly less sensitive while Toledo (pEC50 15.5 µmol/L) and Ly19 (EC50 15.3 µmol/L) were the least sensitive (Figure 2C).

To support the idea that loss of viability was due to on‐target effects on IKKε and TBK1, we determined the sensitivity of Ly10 and Ly03 to a set of nine IKKε/TBK1 inhibitors (described in patent PCT/GB2014/050521) with pIC50 for the purified enzymes varying from 5.8 to 8.6 (for IKKε) and from 6 to 8.3 (for TBK1). There were significant associations between inhibition (pIC50) of TBK1 (Figure 2D) or IKKε (Figure 2E) activity and pEC50 for reduction in cell viability suggesting that loss of viability was indeed through effects on IKKε and TBK1.

In order to detect early changes in phosphorylated proteins Western blots were carried out at time‐points up to 8 hours for Ly10 (Figure 2F) and Ly03 (Figure 2G). DMX3433 caused a reduction in phosphorylated RELA (S536) in both cell lines. We also noted complex effects on STAT3 phosphorylation with an early reduction in STAT3 phosphorylation at tyrosine 705 but an increase at 4 hours in both Ly03 and Ly10 which was sustained in Ly03.

### 3.4 DMX3433 alters the output of growth factors and chemokines

**FIGURE 3:**
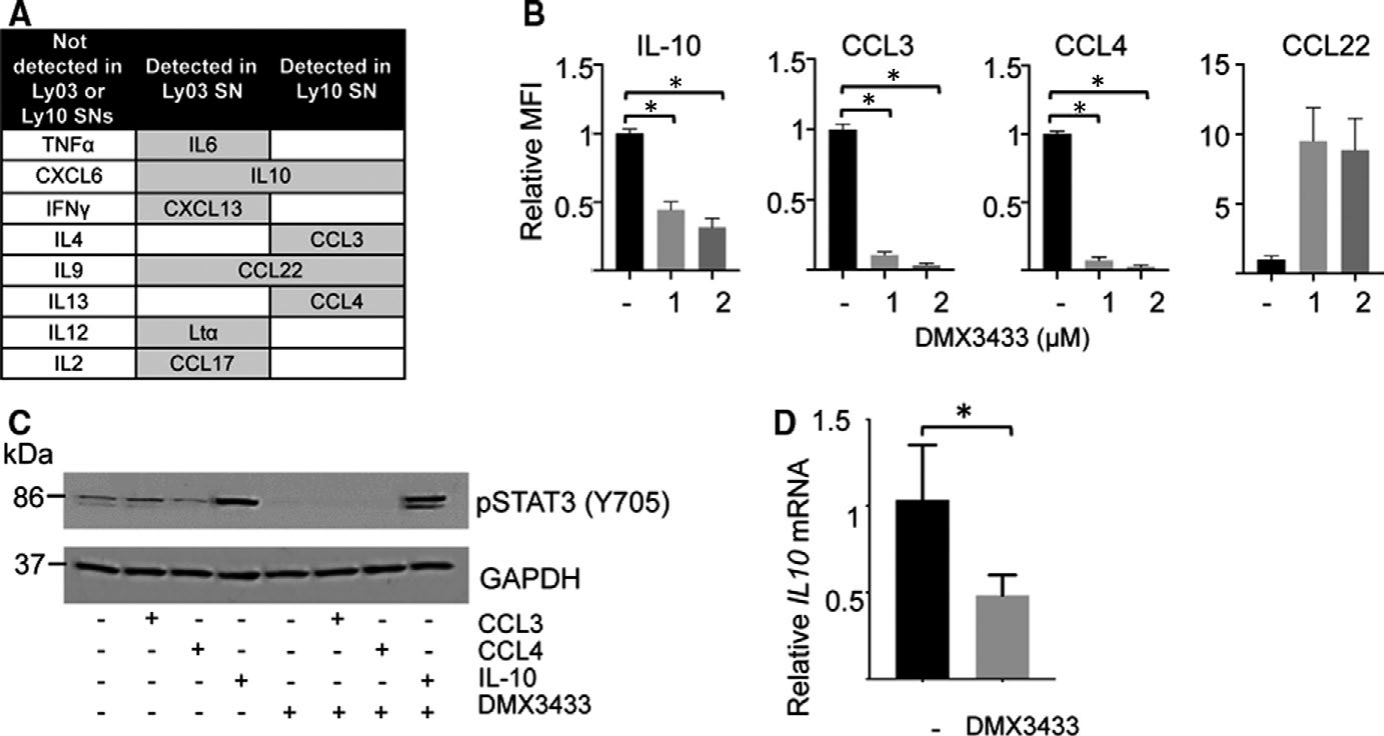
Effects of DMX3433 on expression of RELA, STAT3 and secreted proteins. A, Interleukins and chemokines detected in supernatants from Ly03 and Ly10. Multiplex ELISA was carried out on supernatant from Ly03 and Ly10 after 48 h in culture. Levels of 16 growth factors and chemokines were determined and of these 8 were undetected in either cell line. B, Levels of IL‐10, CCL3, CCL4 and CCl22 determined by multiplex ELISA in response to DMX3433 at concentrations of 1 and 2 µmol/L. Drug treatment causes significant repression (paired t test) of IL‐10 (*P* = .031 at 1 µmol/L and *P* = .032 at 2 µmol/L), CCL3 (*P* = .042 at 1 µmol/L and *P* = .034 at 2 µmol/L), CCL4 (*P* = .032 at 1 µmol/L and *P* = .023 at 2 µmol/L) Mean ± SEM. n = 2. C, Western blots showing effects of added growth factors (CCL3 100 ng/mL, CCL4 100 ng/mL and IL‐10 50 ng/mL) on STAT3 phosphorylation in the presence and absence of DMX3433 in Ly10. Antibodies directed against phosphorylated STAT3 (tyrosine 705) were employed. Molecular weights (kDa) are indicated to the left. GAPDH is a loading control. Representative of 2 separate experiments. Uncropped autorads are in the Appendix. D, Levels of *IL10* mRNA in Ly10 in response to DMX3433 (2 µmol/L). There is a significant reduction (paired t test, *P* = .041) in response to DMX3433. Mean ± SEM. n = 3

NF‐κB drives production of some cytokines, and these, in turn, activate STATs. Using multiplex ELISA to detect a panel of growth factors and chemokines, we investigated the hypothesis that DMX3433 modifies secretion of these proteins in cell lines Ly03 and Ly10, which are the cell lines most sensitive to DMX3433 and, in contrast to other DLBCL cell lines, are known to secrete cytokines^39^ (Figures 3A and S3). 8 of the 15 proteins in our panel were detectable after 48 hours culture without inhibitor, in either Ly03 or Ly10 supernatants but only two of these (IL‐10 and CCL22) were detectable in supernatants from both cell lines. Ly10, which demonstrated the greatest effects of inhibitor to amounts of secreted protein, was used for further studies with DMX3433. This showed significant repression of CCL3 (paired t test, *P* = .042 at 1 µmol/L and *P* = .033 at 2 µmol/L), CCL4 (*P* = .032 at 1 µmol/L and *P* = .023 at 2 µmol/L) and IL‐10 (*P* = .031 at 1 µmol/L and *P* = .032 at 2 µmol/L) (Figure 3B).

**FIGURE 4:**
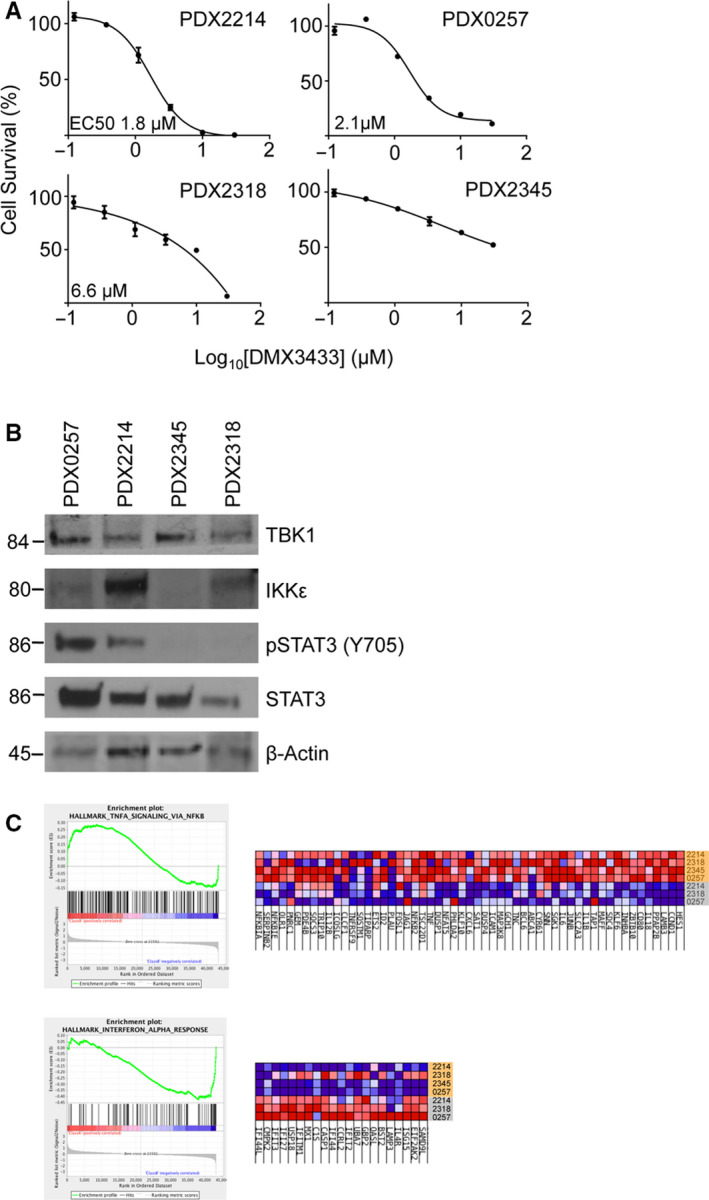
Pre‐clinical testing of DMX3433 in PDX models of DLBCL. A, Dose‐response curves showing effects of DMX3433 on cell survival determined by ATP luminescence on primary human lymphoma cells cultured for 24 h. Four models PDX0257, PDX2214, PDX2345 and PDX2318 were investigated. IC50s (µmol/L) are presented for three of the models but PDX2345 was essentially insensitive. Mean ± SEM. Assays were run in technical triplicate. B, Western blots showing baseline IKKε and TBK1 levels. Phosphorylated (tyrosine 705) and total STAT3 and phosphorylated (serine 536) and total RELA are also shown. Molecular weights (kDa) are indicated to the left. β‐actin is a loading control. Uncropped autorads are in the Appendix. C, Gene set enrichment analysis. Using the entire dataset untreated cells are enriched for the Hallmark gene set "TNFA signalling through NF‐κB" (MSigDB M5890) (FDR = 0.199) and "IFNA response" (M5911) (FDR = 0.005). The leading edge genes for each gene set are presented to the right as a heat map. Lanes highlighted in orange are untreated and lanes in grey are treated. Due to insufficient sample, one treated PDX model (PDX2345) was not included on the treated lanes

IL10 signalling is required for DLBCL survival.^40^ In order to investigate the hypothesis that autocrine IL‐10 signalling is regulated by IKKε/TBK1 and contributes to STAT3 phosphorylation, we added CCL3, CCL4 or IL‐10 to Ly10 cell culture in the presence or absence of DMX3433 (Figure 4A). In the absence of DMX3433 phosphorylation of STAT3 at tyrosine 705 was induced by IL‐10 but not by CCL3 or CCL4. DMX3433 abolished STAT3 phosphorylation, which was restored by IL‐10 suggesting that abolition of tyrosine phosphorylation is mediated principally through this cytokine in Ly10 (Figure 3C). DMX3433 significantly reduced (paired t test, *P* = .041) steady‐state levels of *IL10* mRNA by 8 hours, suggesting that transcriptional repression by the inhibitor is required for reduction in levels of the cytokine in culture supernatants (Figure 3D).

### 3.5 Sensitivity of primary human DLBCL to IKKε/TBK1 inhibitors

Next, we determined the effects of DMX3433 on primary human DLBCL. Four patient‐derived xenografts (PDX) chosen to include two non–GC‐DLBCL (PDX0257 and PDX2345) and two GC‐DLBCL (PDX2214 and PDX2318) were cultured and treated with varying concentrations of drug. PDX0257 and PDX2214 showed the greatest sensitivity to the inhibitor with EC50 of 2.1 and 1.8 µmol/L, respectively, but PDX2318 had an EC50 of 6.6 µmol/L and for PDX2345 EC50 was not reached (Figure 4A). Neither cell‐of‐origin classification nor expression of TBK1 or IKKε appeared to associate with sensitivity to DMX3433 but both sensitive models showed SOCS1 mutations (Table S2) and constitutive pSTAT3 tyrosine 705 expression (Figure 4B). RNA‐seq (obtainable at https://hubase.crownbio.com/) showed that the sensitive models had greater normalized reads for *IL10* (PDX0257 log_2_(fragments per kilobase million, FPKM) 4.43 and PDX2214 log_2_(FPKM) 4.55 vs PDX2345 log_2_(FPKM) 2.04 and PDX2318 log_2_(FPKM) 2.33. Our own gene expression data confirmed the RNA‐seq: In the sensitive PDX models, normalized *IL10* mRNA fluorescence was 10.9 (PDX0257) and 10.5 (PDX2214) but for the resistant models 7.4 (PDX2318) and 7.2 (PDX2345). Again supporting a role for IKKε/TBK1 in regulating IL10 *IL10* mRNA fluorescence reduced from 10.9 to 7.5 (PDX0257) and 10.5 to 7.7 (PDX2214).

Gene expression differences due to drug treatment were determined in both DMX3433 sensitive and resistant PDX models. GSEA showed thirteen Hallmark gene sets in which there was enrichment or depletion of gene expression (Tables S5 and S6) of which ‘TNFα signalling via NF‐κB’ (MSigDB M5890) and ‘IFNA response’ (MSigDB M5911) seemed biologically relevant (Figure 4C). Leading edge analysis showed that, as compared to treated cells, untreated cells were enriched in NF‐κB pathway genes but ‘IFNA response’ genes including type I IFN genes *ISG15, IFIT2, IFIT3, MX1* and *USP18* ^41^ were induced by DMX3433 treatment.

## 4 DISCUSSION

We found that IKKε and TBK1 are expressed in both GC‐ and non–GC‐DLBCL while there were also cases of both subgroups that express no detectable protein. Therefore, expression of these proteins does not segregate with COO subgroups. Recent genetic analysis made possible by comprehensive CRISPR screens suggests that ABC‐ and GC‐DLBCL have some driver mutations in common.^4^ Our results suggest that IKKε and TBK1 are similarly expressed in both COO subgroups.

Analysis of the publicly available gene expression data shows no significant variation in *TBK1* mRNA levels across COO subgroups (Figure 1B) while the immunohistochemistry (Figure 1C, D) shows variation in TBK1 protein expression. This suggests that there are important post‐transcriptional control mechanisms for this protein.

We have shown that small molecule IKKε/TBK1 inhibitors reduce viability of DLBCL cell lines, in particular those expressing RELA and STAT3 and that the effects of a panel of inhibitors correlated with potency of the inhibitors against the purified enzymes, which is consistent with on‐target effects. In addition, we demonstrate that an inhibitor reduced expression of IL‐10 and that exogenous IL‐10 is sufficient to overcome the effect of the inhibitor in reducing STAT3 phosphorylation. Others have shown a MYD88 dependent NF‐κB/IL‐10/JAK2/STAT3 pathway^11^ in DLBCL. MYD88 is downstream of TLRs and, through TRAF6, interacts with TANK and IKKε/TBK1 in normal immune cells^22^, ^42^ (Figure S1). IKKε/TBK1 have been demonstrated to be part of a complex that activates NF‐κB.^22^ There is, therefore, likely to be cross‐talk between the MYD88 and IKKε/TBK1 pathways such that both contribute to NF‐κB signalling. Ly10 and Ly03 (Figure 1F) show constitutive RELA phosphorylation consistent with their known activating MYD88 change p.Leu265Pro and IKKε/TBK1 inhibition reduced RELA phosphorylation. Collectively, our work showed effects of inhibitors on RELA phosphorylation and gene expression, which supports a role for IKKε/TBK1 in contributing to NF‐κB activity in some DLBCL and we note that Ly10, which was used for the gene expression studies has constitutive NF‐κB activity due to MYD88 mutation. Plausibly IKKε/TBK1 are downstream of MYD88 in this cell line. Further work will be needed to establish if MYD88 (p.Leu265Pro) associates with IKKε/TBK1 signalling and sensitivity to IKKε/TBK1 inhibition. However, we found that one cell line sensitive to DMX3433, Pfeiffer, as well as the less sensitive cell lines, did not have detectable phosphorylated RELA (or mutated MYD88). We do not have a clear explanation for why DMX3433 reduced viability of these lines but this may be mediated through effects on other pathways such as interferon signalling and STAT1 or STAT2.^43^, ^44^

Although gene expression profiling supported a role for IKKε/TBK1 in repressing NF‐κB activity we observed that mRNA levels of some type I interferon genes was increased by DMX3433 in primary DLBCL cases. This is consistent with results of a recent study^45^ showing that STAT3 suppresses apoptosis by inhibition of *IRF7*, *STAT1*, *STAT2* and *IRF9* mRNA expression to prevent type I interferon responses in DLBCL. An implication of our work is that one route leading to reduction in viability following inhibition of IKKε/TBK1 is repression of STAT3 phosphorylation, and increased interferon signalling. Proliferation and survival of subgroups of ABC‐DLBCL are dependent on STAT3 signalling^13^, ^46^ and a proportion of GC‐DLBCL cases express pSTAT3.^46^ The mechanism of expression and the clinical importance of pSTAT3 in the GC‐DLBCL subgroup have not been investigated in part because of the lack of representative DLBCL cell lines. STAT3 signalling is associated with proliferation and poor clinical outcome in DLBCL.^47^ Early‐stage clinical trials of STAT3 inhibitors in a variety of disease settings have been reported^48^ and although STAT3 inhibitors do not yet have approvals to treat cancer it is a potential therapeutic target for subgroups of DLBCL.

Biomarkers predicting response to treatment are necessary in order to direct therapeutic agents to patient groups that will benefit the most. We found that CCL3 and CCL4 (as well as IL‐10) were highly expressed by Ly10 and repressed by DMX3433 but levels of another chemokine, CCL22, were induced by the inhibitor. CCL22 is repressed by type I interferon responses^49^ and it is, therefore, possible that the increase we observed with DMX3433 is an on‐target effect. Circulating levels of CCL3 and CCL4 associate with clinical outcome in ABC‐DLBCL but not in GC‐DLBCL.^50^ Higher levels of circulating IL‐10 correlate with shorter event‐free survival and higher lactose dehydrogenase (LDH) levels^51^ and the genes for the IL10 receptors (IL10RA and IL10RB) are amplified in 21% (IL10RA) and 10% (IL10RB) of DLBCL patients, respectively, and associated with higher IL10RA and IL10RB mRNA than normal subjects.^40^ Levels of CCL3, CCL4 or IL‐10 may, therefore, be suitable biomarkers predicting sensitivity to IKKε/TBK1 inhibitors. The most sensitive PDX models, PDX0257 and PDX2214, show SOCS1 mutations, which could enhance STAT3 signalling. The clinical effects of SOCS1 mutations are controversial but a recent retrospective study of elderly patients who had received R‐CHOP suggested that the presence of mutations confers a poor prognosis. One possibility prompted by our work is that SOCS1 mutation might be a marker for response to IKKε/TBK1 inhibitors.

A limitation of our work is that we focused on the Ly10 DLBCL cell line and used relatively few PDX models. Limitations on the amount of material available from the PDX models also meant that we could not carry out a more extensive analysis. Ly10 is favourable because it produces relatively large amounts of growth factors and has been used by others for this purpose^39^ but it is clearly not representative of DLBCL. In view of the heterogeneity of DLBCL, larger studies using both cell lines and primary cells will be required.

Data presented here demonstrate that the innate immunity components, IKKε and TBK1 are expressed in primary DLBCL but in a manner that is not strictly dependent on COO subgroup. IKKε/TBK1 inhibitors suppress survival of some DLBCL cell lines and focusing on the most sensitive cell line, Ly10, we show that an IKKε/TBK1 inhibitor suppresses RELA phosphorylation, IL‐10 production and STAT3 phosphorylation. We observed that both the sensitive DLBCL cell lines and the sensitive PDX models had high STAT3 expression, a previously defined characteristic of some DLBCL in both GC‐ and ABC‐DLBCL subgroups.^13^, ^46^ Further work will be necessary to characterize in detail the subgroups of DLBCL that are most sensitive to inhibition of IKKε/TBK1.

## CONFLICT OF INTEREST

The authors declare no competing financial interests other than that TP and KLC are employees of Domainex Ltd. and TP is an inventor of patent PCT/GB2014/050521 that claims *inter alia* DMX3433 as an IKKε/TBK1 inhibitor.

## AUTHOR CONTRIBUTIONS

**Matthew Carr:** Formal analysis (equal); Investigation (lead); Methodology (equal); Writing‐review & editing (equal). **Sami Mamand:** Formal analysis (supporting); Investigation (supporting); Writing‐review & editing (equal). **Kathryn Louise Chapman:** Formal analysis (equal); Resources (equal); Supervision (equal); Writing‐review & editing (equal). **Trevor Perrior:** Funding acquisition (equal); Project administration (equal); Resources (equal); Supervision (equal); Writing‐review & editing (equal). **Simon Wagner:** Conceptualization (equal); Funding acquisition (equal); Project administration (equal); Supervision (equal); Writing‐original draft (equal); Writing‐review & editing (equal).

## ETHICS APPROVAL AND CONSENT TO PARTICIPATE

All animal studies were conducted at Crown Bioscience HuPrime SPF animal facility (CrownBio, Beijing, China) under sterile conditions and were in strict accordance with the Guide for the Care and Use of Laboratory Animals of the National Institutes of Health. Protocols for all studies were approved by the Committee on the Ethics of Animal Experiments of Crown Bioscience, Inc (Crown Bioscience IACUC Committee). The patient‐derived xenograft models were obtained from Crown Bioscience.

## Supporting information

Appendix S1

Appendix S2

## Data Availability

The data that support the findings of this study are openly available in Gene Expression Omnibus (GEO) at https://www.ncbi.nlm.nih.gov/geo/, reference number GSE121159.
