# Supplementary material for: IKKε and TBK1 in diffuse large B‐cell lymphoma: A possible mechanism of action of an IKKε/TBK1 inhibitor to repress NF‐κB and IL‐10 signalling
Source: J Cell Mol Med. 2020 Aug 28;24(19):11573–82. doi: 10.1111/jcmm.15774 (PMC7576278; doi:10.1111/jcmm.15774)

Additional File 1

Carr M, Chapman K, Perrior T and Wagner SD

Supplementary Table 1, Additional File 1

Results of analysis of tissue microarray 1 (OD-CT-LyMly02-001). Position on the microarray and age and sex of the patient are indicated in the left-hand columns. Staining with anti-CD10, anti-BCL6 and anti-MUM1 antibodies was carried out to determine GC or non-GC assignment. Slides were assessed by two independent observers. Positive (+) and negative (-) staining are indicated. In some cases staining was indeterminate (?). Results of IKKε and TBK1 immunofluorescence are also presented. Tissue sections that could not be assessed due to poor quality are indicated in gray.

Position	Age	Sex	CD10	BCL6	MUM1	GC/non-GC	IKKε	TBK1
A1	72	F	-	-	+	Non-GC	+	+
A2	74	F	+	?	-	GC	+	+
A3	50	M	-	+	-	GC	+	-
A4	60	M	-	-	-	Non-GC	+	+
A5	38	M	-	-	+	Non-GC	+	+
A6	64	F	-	-	-	Non-GC	+	+
B1	73	M	-	+	-	GC	-	-
B2	55	M	-	+	-	GC	-	-
B3	53	M	-	-	-		-	-
B4	73	F	+	+	-	GC	+	-
B5	50	F	-	-	-		-	-
B6	81	F	-	-	-	Non-GC	+	+
C1	72	M	+	-	-	GC	-	-
C2	55	M	+	+	+	GC	-	-
C3	58	M	-	+	-	GC	-	-
C4	81	F	+	-	-	GC	-	-
C5	57	M	+	+	-	GC	-	-
C6	61	M	-	+	-	GC	-	-
D1	52	F	-	+	-	GC		+
D2	65	M	?	+	-	GC	-	+
D3	78	M	-	-	-	Non-GC		+
D4	32	F	-	-	-		-	-
D5	60	M	+	+	?	GC	-	-
D6	73	M	-	-	-	Non-GC	+	-
E1	47	M	+	+	+	GC	+	+
E2	65	F	-	-	+	Non-GC	-	+
E3	64	M	+	+	-	GC	-	+
E4	75	M	-	-	+	Non-GC	+	+
E5	58	M	-	-	+	Non-GC	+	+
E6	56	M	-	-	+	Non-GC	+	+

Supplementary Table 2, Additional File 1

Results of analysis of tissue microarray 2 (Ly1001c). Position on the microarray and number assigned to the patient are indicated in the left-hand columns. GC or non-GC status is given to the right. Slides were assessed by two independent observers. Results of IKKε and TBK1 immunofluorescence are also indicated as being positive (+) or negative (-).

Position	Number	IKKε	TBK1	GC/Non-GC
D4	#34	+	+	Non-GC
D5	#35	-	+	Non-GC
D6	#36	-	+	Non-GC
D7	#37	+	-	GC
D8	#38	+	+	Non-GC
D9	#39	-	-	GC
D10	#40	-	-	Non-GC
E1	#41	+	-	Non-GC
E2	#42	-	-	Non-GC
E3	#43	-	-	GC
E4	#44	+	+	Non-GC
E5	#45	-	-	GC
E6	#46	-	+	Non-GC
E7	#47	-	+	Non-GC
E8	#48	-	-	Non-GC
E9	#49	-	-	GC
E10	#50	+	+	Non-GC
F2	#52	-	+	GC
F3	#53	+	-	Non-GC
F4	#54	-	+	Non-GC
F5	#55	-	-	GC
F6	#56	-	+	GC
F7	#57	-	-	GC
F8	#58	+	+	Non-GC
F9	#59	-	+	Non-GC
F10	#60	+	+	GC
G1	#61	+	-	Non-GC
G2	#62	+	-	GC
G3	#63	-	-	GC
G4	#64	+	+	Non-GC
G5	#65	-	-	GC
G6	#66	+	+	Non-GC
G7	#67	-	-	GC
G8	#68	-	-	Non-GC
G9	#69	-	-	Non-GC
G10	#70	-	-	Non-GC
H1	#71	-	-	GC
H2	#72	+	-	Non-GC
H3	#73	+	-	Non-GC
H4	#74	+	+	Non-GC
H5	#75	-	-	GC
H6	#76	+	+	Non-GC
H7	#77	-	-	Non-GC
H8	#78	+	-	Non-GC
H9	#79	-	-	Non-GC
H10	#80	-	-	Non-GC
I1	#81	+	-	GC
I2	#82	-	-	Non-GC
I3	#83	+	-	Non-GC
I4	#84	+	-	Non-GC
I5	#85	-	-	GC
I6	#86	-	-	GC
I7	#87	-	-	GC
I8	#88	+	-	Non-GC
I9	#89	+	+	GC
I10	#90	+	+	Non-GC
J2	#92	+	-	GC
J3	#93	+	-	Non-GC
J4	#94	+	-	GC
J5	#95	-	-	GC
J6	#96	-	+	Non-GC
J7	#97	+	-	Non-GC
J8	#98	+	-	GC
J9	#99	-	-	Non-GC
J10	#100	-	-	GC

Supplementary Table 3, Additional File 1

Characteristics of patients by TBK1(> median or < median) expression. TBK1 is represented by Affymetrix probe 218520_at on the gene expression microarray (Lenz et al. 2008)[30].

		TBK1 Expression	
		<Median	>Median
DLBCL	GC	85	82
	ABC	78	76
	Unclassified	25	34
Performance Score	0	46	43
	1	87	105
	2	36	27
	3	15	12
	4	4	0
Clinical Stage	1	33	31
	2	61	55
	3	39	40
	4	55	63
Extranodal sites of disease	0	129	144
	1	43	48
	2	4	0
	3	6	0
	4	0	0
	5	1	0
LDH	>ULN	106	96
	x2ULN	28	51
Age	Range	14-92	18-88
	Median	62	66

Supplementary Table 4, Additional File 1

Known characteristics of the four PDX models used, data taken from Crown Bioscience (SanDiego, CA, USA) HuBase. The mutated gene is indicated (bold) together with the mutated amino acid residue and its position.

PDX Model	GC/Non-GC status	Genetics
0257	Non-GC	MYD88 (L273P), KMT2D (L208fs), PIM1 (L2V, K29N, S75F, L80M, L106I, E141Q, G45D), PRDM1 (S429N), SOCS1 (C111Y), TNFRSF14 (K17R), BCL6 translocation
2214	GC	CREBBP (R1730C), KMTD (P2382S, M3349V), PIM1 (T23I), SOCS1 (M1V, A17P, P25S), TP53 (P72R), MYC translocation
2345	Non-GC	CARD11 (A687V), EZH2 (D185H), TNFAIP3 (F127C, I194T), PRDM1 (G74S), TNFRS14 (K17R), TP53 (P72R)
2318	GC	TNFSR14 (K17R), TP53 (P72R)

Supplementary Table 5, Additional File 1

Contingency table of tissue microarray results showing IKKe and TBK1 expression in cases of DLBCL. Applying a chi-square test, the chisquare value = 9.65 and *P*=0.022.

	IKKε+TBK1+	IKKε+TBK1-	IKKε-TBK1+	IKKε-TBK1-	
GC	2	6	2	16	26
Non-GC	11	11	7	10	39

Supplementary Table 6, Additional File 1

Hallmark gene sets identified through GSEA of the treated PDX data set. The thirteen gene sets all have normalised enrichment scores <-1 or >1, P-value <0.05 and FDR <0.25. The four gene sets highlighted in grey appear to have the most relevance and leading edge analyses are presented: "TNFα signalling via NF-κB" and "IFNA response" are shown in Figure 5C.

Hallmark Gene Set	Normalised Enrichment Score	Nominal P-value	FDR q-value
Allograft Rejection	1.77	<0.001	0.002
Hypoxia	1.52	<0.001	0.141
Apical Junction	1.50	0.006	0.116
UV Response Downregulated	1.46	0.004	0.136
Androgen Response	1.39	0.043	0.179
Estrogen Response	1.36	0.018	0.202
Inflammatory Response	1.34	0.035	0.22
IL2/STAT5 Signalling	1.31	0.021	0.215
TNFα Signalling via NF-κB	1.31	0.021	0.199
Oxidative phosphorylation	1.29	0.044	0.2
Epithelial Mesenchymal Transition	1.28	0.036	0.196
UV Response Upregulated	1.27	0.048	0.193
IFNA Response	-1.76	<0.001	0.005

Supplementary Table 7, Additional File 1

Gene List for significantly different genes in PDX models following DMX3433 treatment. Fold change is presented to the right, with down-regulated genes highlighted in blue and up-regulated genes in red. Expected score, observed score, numerator and denominator are also presented.

Gene name	Accessions	Expected score (dExp)	Observed score (d)	Numerator (r)	Denominator (s + s0)	Fold change (untreated vs treated)
HLA-DRB6	ref|NR_001298|ens|ENST00000437650|ens|ENST00000437183|ens|ENST00000411500	0.69250524	-6.0541134	-7.448064	1.2302485	0.003986448
NGFRAP1	ref|NM_014380|ref|NM_206915|ref|NM_206917|ref|NM_001282674	0.13777603	-4.955232	-9.4411335	1.9052858	0.007832157
OAT	ref|NM_000274|ref|NM_001171814|ens|ENST00000368845|ens|ENST00000539214	-0.42711043	-10.412256	-6.8296976	0.6559287	0.008128105
BZW2	ref|NM_001159767|ref|NM_014038|ref|NR_027624|ens|ENST00000436868	-0.23832299	-6.976292	-7.3703356	1.0564833	0.009416273
LOC100128108	ref|XM_006709989|ref|XM_006720812|ref|XM_006726585|ref|XM_006709990	1.4540544	-8.703404	-6.5037103	0.7472605	0.009826303
PAX8-AS1	ref|NR_047570|linc|lnc-PSD4-1:4|gb|AK126431|gb|AK056052	0.9089403	-5.731176	-6.136895	1.0707916	0.011258191
BZW2	ref|NM_014038|ref|NM_001159767|ref|NR_027624|ens|ENST00000436868	0.12658124	-5.1819177	-7.757532	1.4970388	0.012361586
TANC1	ref|NM_033394|ref|NM_001145909|ens|ENST00000470074|ens|ENST00000454300	0.4425247	-13.821071	-6.267383	0.45346582	0.012637749
RGS13	ref|NM_002927|ref|NM_144766|ens|ENST00000391995|ens|ENST00000462955	-0.0769816	-5.4684925	-6.0562477	1.1074803	0.013509267
EPB41L4B	ref|NM_018424|ens|ENST00000374557|gb|AK000388|gb|AF153418	0.25864816	-14.808917	-6.0600777	0.40921816	0.014920611
COL9A3	ref|NM_001853|ens|ENST00000466192|ens|ENST00000343916|gb|BC011705	-0.12108333	-5.2622437	-6.0246463	1.1448816	0.016549164
WBP5	ref|NM_016303|ref|NM_001006612|ref|NM_001006613|ref|NM_001006614	0.20630205	-6.341204	-6.4480085	1.016843	0.017153626
NANOS1	ref|NM_199461|ens|ENST00000340087|ens|ENST00000425699|gb|AF275269	0.66347855	-7.2370815	-5.29869	0.73215836	0.023540646
TMEM2	ref|NM_013390|ref|NM_001135820|ens|ENST00000474495|ens|ENST00000542935	0.122671515	-6.8060956	-5.224328	0.76759547	0.024705239
ZNF177	ref|NM_003451|ref|NM_001172651|ref|NM_001202425|ref|NM_001172650	-0.056044735	-6.561065	-5.20105	0.79271424	0.02525807
C1orf21	ref|NM_030806|ens|ENST00000367514|ens|ENST00000235307|gb|AK312062	0.38429293	-8.157888	-4.945039	0.60616654	0.03064756
MVB12B	ref|NM_033446|ens|ENST00000361171|ens|ENST00000485886|ref|XM_005252297	0.44425797	-5.523779	-5.034725	0.9114639	0.035319015
PPAP2B	ref|NM_003713|ens|ENST00000371250|gb|AF480883|tc|THC2585233	-0.045502238	-9.324073	-4.8154707	0.5164557	0.035554193
GPR171	ref|NM_013308|ens|ENST00000617554|ens|ENST00000309180|ref|XM_005247403	0.11935581	-5.120761	-4.3056984	0.84083176	0.040272098
LOC100128242	gb|XR_110532|tc|THC2635860|tc|THC2605246	1.4987639	-8.325114	-4.535375	0.54478234	0.04140975
XLOC_l2_015722	linc|TCONS_l2_00030483	1.3095869	-5.3480735	-4.468046	0.83544964	0.041783236
LAMB3	ref|NM_001017402|ref|NM_000228|ref|NM_001127641|ens|ENST00000367030	-0.34077552	-5.41677	-4.469247	0.825076	0.04273167
CCND1	ref|NM_053056|ens|ENST00000227507|ref|XM_006718653|gb|M73554	0.45578006	-6.007808	-4.2209454	0.7025766	0.0507324
ZNF185	ref|NM_001178106|ref|NM_001178107|ref|NM_001178108|ref|NM_007150	-0.22086945	-6.316081	-4.2213387	0.6683478	0.05799075
CTSV	ref|NM_001333|ref|NM_001201575|ens|ENST00000259470|ens|ENST00000538255	-0.14094153	-5.2736673	-4.0253	0.7632829	0.060051713
ZBTB8A	ref|NM_001040441|ref|NM_001291496|ref|NR_111980|ens|ENST00000480336	-0.30962974	-7.330445	-4.0486627	0.5523079	0.06284407
ZNF571	ref|NM_016536|ref|NM_001290314|ens|ENST00000358744|ens|ENST00000451802	0.2141461	-4.8374267	-3.9474676	0.81602633	0.06344262
VSTM2L	ref|NM_080607|ens|ENST00000373461|gb|AK056177|gb|AL834410	0.4632335	-10.926288	-3.903103	0.35722134	0.06652255
HES1	ref|NM_005524|ens|ENST00000476918|ens|ENST00000232424|gb|AK300482	0.026889302	-7.051898	-3.7693992	0.53452265	0.069763705
XLOC_l2_004817	linc|TCONS_l2_00008876|gb|AK127420|tc|THC2483012	1.1456883	-5.572576	-3.86513	0.69359845	0.070021465
RAB26	ref|NM_014353|ens|ENST00000564426|ens|ENST00000210187|gb|AK308560	0.13654827	-7.0304117	-3.8225513	0.54371655	0.073032565
DAB2IP	ref|NM_032552|ens|ENST00000259371|ens|ENST00000373782|ens|ENST00000408936	0.41883275	-5.9955006	-3.9373758	0.6567218	0.07306519
ALDH2	ref|NM_000690|ref|NM_001204889|ens|ENST00000261733|gb|AK223373	-0.40952644	-5.4263825	-3.8775449	0.7145727	0.078158274
FGL2	ref|NM_006682|ens|ENST00000248598|gb|CR626993|tc|THC2610602	0.073959984	-6.6256204	-3.65396	0.5514895	0.080222346
IZUMO4	ref|NM_001031735|ref|NM_001039846|ens|ENST00000481489|ens|ENST00000590985	-0.32728103	-5.0771747	-3.6365712	0.7162588	0.08506301
WFS1	ref|NM_006005|ref|NM_001145853|ens|ENST00000503569|ens|ENST00000507765	0.04615267	-5.6450877	-3.5366504	0.62650055	0.08950899
ZNF10	ref|NM_015394|ens|ENST00000426665|ens|ENST00000248211|gb|AK314784	0.17804244	-8.292993	-3.474423	0.41895887	0.08989479
ZNF28	ref|NM_006969|ref|NR_036599|ref|NR_036600|ens|ENST00000457749	0.0850408	-6.192596	-3.4498577	0.5570939	0.09033825
SLC27A2	ref|NM_003645|ref|NM_001159629|ens|ENST00000380902|ens|ENST00000267842	-0.04810635	-8.733956	-3.4710886	0.39742455	0.09096816
DLK2	ref|NM_206539|ref|NM_001286655|ref|NM_023932|ref|NM_001286656	0.67294294	-6.1458855	-3.510931	0.5712653	0.09168304
HCG23	ref|NR_044996|linc|lnc-HLA-DRA-1:1|tc|THC2636745|tc|THC2718499	0.878767	-6.850795	-3.460706	0.50515395	0.09566069
ZNF563	ref|NM_145276|ens|ENST00000293725|ref|XM_005259751|ref|XM_005259748	0.51590794	-7.0002446	-3.394183	0.48486632	0.09635101
ENST00000617217	ens|ENST00000617217|ens|ENST00000610645	-0.4490303	-5.9433155	-3.2436855	0.54577035	0.1006513
ENST00000453953	ens|ENST00000453953|linc|lnc-ASMT-4:1|linc|TCONS_00016961|linc|TCONS_00017603	-0.7458464	-6.2320876	-3.1900754	0.5118791	0.105848886
SLC26A9	ref|NM_052934|ref|NM_134325|ens|ENST00000367134|ens|ENST00000491127	0.4516701	-5.5394726	-3.1711829	0.5724702	0.107583754
GK	ens|ENST00000378938	-0.9887323	-5.3612366	-3.177529	0.5926859	0.10801281
ENO2	ref|NM_001975|ens|ENST00000535366|ens|ENST00000541477|ens|ENST00000229277	-0.11634903	-5.405869	-2.9123383	0.53873634	0.13155599
RTN3	ens|ENST00000338850|ens|ENST00000538995|tc|THC2690807|tc|THC2624123	-1.0336533	-6.1281734	-2.9373443	0.47931808	0.13161409
THC2539168	tc|THC2539168	1.3341184	-5.551708	-2.820846	0.50810415	0.13640998
ENST00000597683	ens|ENST00000597683|ens|ENST00000594934|linc|lnc-ZNF254-1:2|linc|lnc-ZNF254-1:1	-0.46920437	-5.0632253	-2.8784134	0.568494	0.13853358
SPATA24	ref|NM_194296|ens|ENST00000450845|ref|XM_006714566|ref|XM_005271916	0.63999873	-5.1570096	-2.8386867	0.5504521	0.14030375
COPZ2	ref|NM_016429|ens|ENST00000612370|ens|ENST00000584955|ens|ENST00000585182	0.21039689	-8.961029	-2.8138795	0.31401297	0.14225385
ZNF540	ref|NM_152606|ref|NM_001172225|ref|NM_001172226|ens|ENST00000589285	0.5407232	-8.05765	-2.723476	0.3379988	0.15041964
GGTLC2	ref|NM_001282879|ref|NM_199127|ref|NM_001288833|ref|NM_013421	-0.16419788	-4.8055916	-2.641961	0.54976815	0.1534097
NPEPL1	ref|NM_024663|ref|NM_001204872|ref|NM_001204873|ref|NR_037945	0.35784194	-4.960142	-2.5839195	0.5209366	0.16487488
FZD6	ref|NM_003506|ref|NM_001164615|ref|NM_001164616|ens|ENST00000522566	-0.053805295	-5.826966	-2.5633216	0.43990675	0.17337336
SEC14L1	ref|NM_003003|ref|NM_001204410|ref|NM_001143998|ref|NM_001143999	-0.07375648	-5.4608326	-2.4759378	0.45339933	0.17803082
CORO1B	ref|NM_001018070|ref|NM_020441|ens|ENST00000545736|ens|ENST00000453768	-0.33784893	-4.787349	-2.4649332	0.51488477	0.1798145
PLEKHH1	ref|NM_020715|ens|ENST00000559168|ens|ENST00000557971|ens|ENST00000558214	0.29409885	-7.917587	-2.46103	0.31083083	0.18093576
DUSP13	ref|NM_001007271|ref|NM_001007273|ref|NM_001007272|ref|NM_016364	-0.36316213	-5.051703	-2.4821882	0.49135673	0.18661933
ZNF772	ref|NM_001024596|ref|NM_001144068|ens|ENST00000610548|ens|ENST00000615214	-0.33549613	-5.813289	-2.2737799	0.39113483	0.20551583
DGAT2	ref|NM_032564|ref|NM_001253891|ens|ENST00000603363|ens|ENST00000228027	0.4193252	-5.770708	-2.2520366	0.39025307	0.210023
CUL7	ref|NM_014780|ref|NM_001168370|ens|ENST00000535468|ens|ENST00000265348	0.1532988	-4.9287543	-2.2208147	0.45058337	0.21120834
BCL7A	ref|NM_020993|ref|NM_001024808|ens|ENST00000538010|gb|X89984	0.3050089	-5.1823654	-2.18441	0.4215083	0.22252312
MTL5	ref|NM_001039656|ens|ENST00000443940|gb|AK128308|gb|AK074817	-0.31479383	-5.4736915	-2.1470385	0.3922469	0.22348407
ENST00000534728	ens|ENST00000534728|linc|TCONS_l2_00004831|gb|AK128036|linc|TCONS_l2_00004830	-0.58418596	-7.877059	-2.1578095	0.27393594	0.22393538
PIK3C2B	ref|NM_002646|ens|ENST00000424712|ens|ENST00000367187|ref|XM_005245257	-0.08814828	-6.207716	-2.1554356	0.34721878	0.224699
ACOT9	ref|NM_001037171|ref|NM_001033583|ens|ENST00000449612|ens|ENST00000494361	-0.32085207	-4.9476233	-2.130725	0.43065625	0.2251833
SEC14L1	ref|NM_001204410|ref|NM_001143998|ref|NM_001143999|ref|NM_003003	-0.20285474	-6.3963776	-2.0906029	0.32684168	0.23520501
FAM110A	ref|NM_001042353|ref|NM_001289145|ref|NM_001289146|ref|NM_031424	-0.30780342	-5.4798326	-2.098174	0.38289016	0.23651303
ENST00000427083	ens|ENST00000427083	-0.8588177	-5.377107	-1.9218249	0.3574087	0.26287654
GNAS	ref|NM_001077489|ref|NM_001077490|ref|NM_080425|ref|NM_016592	-0.299439	-5.0199404	-1.7829838	0.35518026	0.2923128
RNASEL	ref|NM_021133|ens|ENST00000367559|gb|BC090934|tc|THC2487723	0.3098604	-5.785814	-1.7223887	0.2976917	0.30227068
TNFAIP6	ref|NM_007115|ens|ENST00000460812|ens|ENST00000243347|gb|AJ419936	0.09002295	5.2806478	2.499771	0.47338343	5.7690287
ENST00000454116	ens|ENST00000454116|ens|ENST00000424993|ens|ENST00000438540|ens|ENST00000510867	-0.74550086	6.2228994	2.531146	0.40674707	5.8126793
CYP4V2	ref|NM_207352|ens|ENST00000507209|ens|ENST00000502665|ref|XM_005262935	0.67972195	5.4666796	2.7094944	0.49563804	6.4231696
HDX	ref|NM_144657|ref|NM_001177479|ref|NM_001177478|ens|ENST00000373177	0.5020065	5.31168	2.7459698	0.51696825	6.936426
USP41	ens|ENST00000454608|ens|ENST00000486536|ref|XM_006726833|ref|XM_006724407	-0.7428457	5.31454	2.838113	0.534028	6.9846725
LINC01013	ens|ENST00000458028|linc|lnc-ENPP1-4:9|linc|lnc-ENPP1-4:8|linc|TCONS_00011190	-0.7293333	7.2795334	2.88161	0.3958509	7.478504
LRRK2	gb|AK127729|tc|THC2486890	-1.1702874	5.42555	3.16466	0.5832883	8.466957
PTPRK	ref|NM_002844|ref|NM_001291981|ref|NM_001135648|ref|NM_001291984	-0.08056853	5.3974233	3.5267797	0.65341914	10.907997
EPB41L4A	ref|NM_022140|ens|ENST00000515047|ens|ENST00000507810|ens|ENST00000261486	0.32703352	7.453211	3.40165	0.4564006	10.943115
THC2686816	tc|THC2686816	1.3951015	9.09769	3.4597328	0.38028696	11.022622
SSX4B	ref|NM_001034832|ref|NM_001040612|ref|NM_005636|ref|NM_175729	-0.3225909	9.607048	3.4981277	0.364121	11.289821
XLOC_l2_004951	linc|TCONS_l2_00009129	1.1477847	7.1623144	3.479104	0.48575136	11.5992985
THC2650820	tc|THC2650820	1.3765107	7.532567	3.509476	0.46590704	11.684649
LINC00477	ref|NR_029451|ens|ENST00000483544|linc|lnc-BCAT1-1:1|gb|AK057456	0.8033398	6.0126667	3.5491233	0.5902744	12.050297
A2MP1	ref|NR_040112|ens|ENST00000543404|linc|lnc-PZP-1:1|tc|THC2644733	0.8763154	5.5711875	3.7472687	0.67261577	12.7509775
ENST00000556060	ens|ENST00000556060|ref|XR_424337|ref|XR_429061|ref|XR_432805	-0.5497063	8.023434	3.671163	0.45755512	12.805765
CCDC181	ref|NM_021179|ref|NM_001300968|ref|NM_001300969|ens|ENST00000456107	0.31155285	7.100389	3.7062845	0.5219833	13.7584715
ZNF711	ref|NM_021998|ens|ENST00000276123|ens|ENST00000373165|ref|XM_006724687	0.32235497	6.032961	3.942583	0.6535072	17.45179
PERP	ref|NM_022121|ens|ENST00000421351|gb|AK074585|gb|AK075082	0.32608867	5.9753256	4.1045017	0.6869085	17.786291
ITM2C	ref|NM_030926|ref|NM_001287241|ref|NM_001287240|ref|NM_001012514	0.38660866	7.3204894	4.254988	0.5812437	19.515285
EGR1	ref|NM_001964|ens|ENST00000239938|gb|M80583|gb|M62829	-0.116842546	6.585296	4.4371643	0.67379874	22.827417
MAGEA12	ref|NM_005367|ref|NM_001166386|ref|NM_001166387|ens|ENST00000393900	0.019889269	5.861811	4.8118715	0.82088476	23.91025
XCL1	ref|NM_002995|ref|NM_003175|ens|ENST00000367818|ens|ENST00000367819	-0.0740893	9.623984	4.6640415	0.4846269	25.94661
CST5	ref|NM_001900|ens|ENST00000304710|gb|AB590893|gb|BC069514	-0.11938617	7.301627	4.731806	0.6480481	28.236906
GAS2L3	ref|NM_174942|ref|NM_001303131|ref|NM_001303130|ens|ENST00000266754	0.5895099	9.366558	5.032991	0.53733623	34.686733
MKRN3	ref|NM_005664|ens|ENST00000314520|gb|BC044639|gb|BC013970	0.03291065	16.299408	5.1796017	0.31777853	36.1421
CASC9	ref|NR_103848|ref|NR_103849|ref|NR_103850|ens|ENST00000523313	0.9295241	12.701431	5.383928	0.42388356	41.451485
XLOC_l2_006399	linc|TCONS_l2_00011955	1.1627076	10.421616	5.3225636	0.5107235	41.45313
PIEZO2	ref|NM_022068|ens|ENST00000538948|ens|ENST00000383408|gb|AK302764	0.32399344	19.343405	5.417257	0.28005704	42.677284
IFI44L	ref|NM_006820|ens|ENST00000476521|ens|ENST00000486882|ens|ENST00000370751	0.07930994	7.2829986	5.254736	0.7215072	43.89676
CST3	ref|NM_000099|ens|ENST00000398409|ens|ENST00000376925|gb|CR542018	-0.43508947	12.216241	6.332365	0.51835626	84.56265
ENST00000390624	ens|ENST00000390624|gb|Z18824|gb|KJ423377|gb|EF175424	-0.96563387	5.793541	6.020211	1.0391246	91.461105
HPDL	ref|NM_032756|ens|ENST00000334815|gb|BC007293|tc|THC2464533	0.42432627	5.6033416	6.868838	1.2258468	127.70899
CXorf57	ref|NM_018015|ref|NM_001184782|ens|ENST00000372544|ens|ENST00000421550	0.24166639	12.328476	7.2077637	0.58464354	158.48029
BBOX1	ref|NM_003986|ens|ENST00000525090|ens|ENST00000529202|ens|ENST00000263182	-0.034810178	5.4385233	6.6791725	1.2281225	167.29807
ENST00000558425	ens|ENST00000558425|ens|ENST00000565407|ens|ENST00000354689|linc|TCONS_l2_00010131	-0.5423408	10.236918	7.226892	0.7059636	171.03789
MAGEA1	ref|NM_004988|ens|ENST00000356661|gb|BC017555|tc|THC2476756	0.004460861	6.8895326	8.490363	1.2323569	196.98857
ENST00000426099	ens|ENST00000426099|ens|ENST00000425181|ens|ENST00000565524|ref|XM_006726634	-0.8641102	10.320354	8.356282	0.80968946	385.03122
	ref|NM_139250|ref|NM_001327|ens|ENST00000328435|ens|ENST00000599837	0.49657655	6.74753	11.618609	1.7219056	7118.4834

Supplementary Figure 1, Additional File 1

IKKε/TBK1 signalling pathways (adapted from Barton GM and Medzhitov R. 2003. Nature Immunology. 4:432-433). Signalling through TLR3 and TLR4 to TIR domain containing adaptor inducing interferon β (TRIF) activates IKK activates IKKε and TBK1 to drive interferon regulatory factor 3 (IRF3) and interferon-α/β (IFN α/β) responses. There is cross-talk with signalling pathways from TLR7 and TLR9, through MYD88 and TRAF6 that activates NF-κB. IKKε/TBK1 interact with TRAF family member associated NF-κB activator (TANK), which also associates with TRAF6. TANK, therefore, links the MYD88 pathway to IKKε/TBK1 signalling. In the diagram the pathways, which are the focus of this work are highlighted in blue and linked by black lines. Secondary pathways are indicated by grey shapes and grey lines.

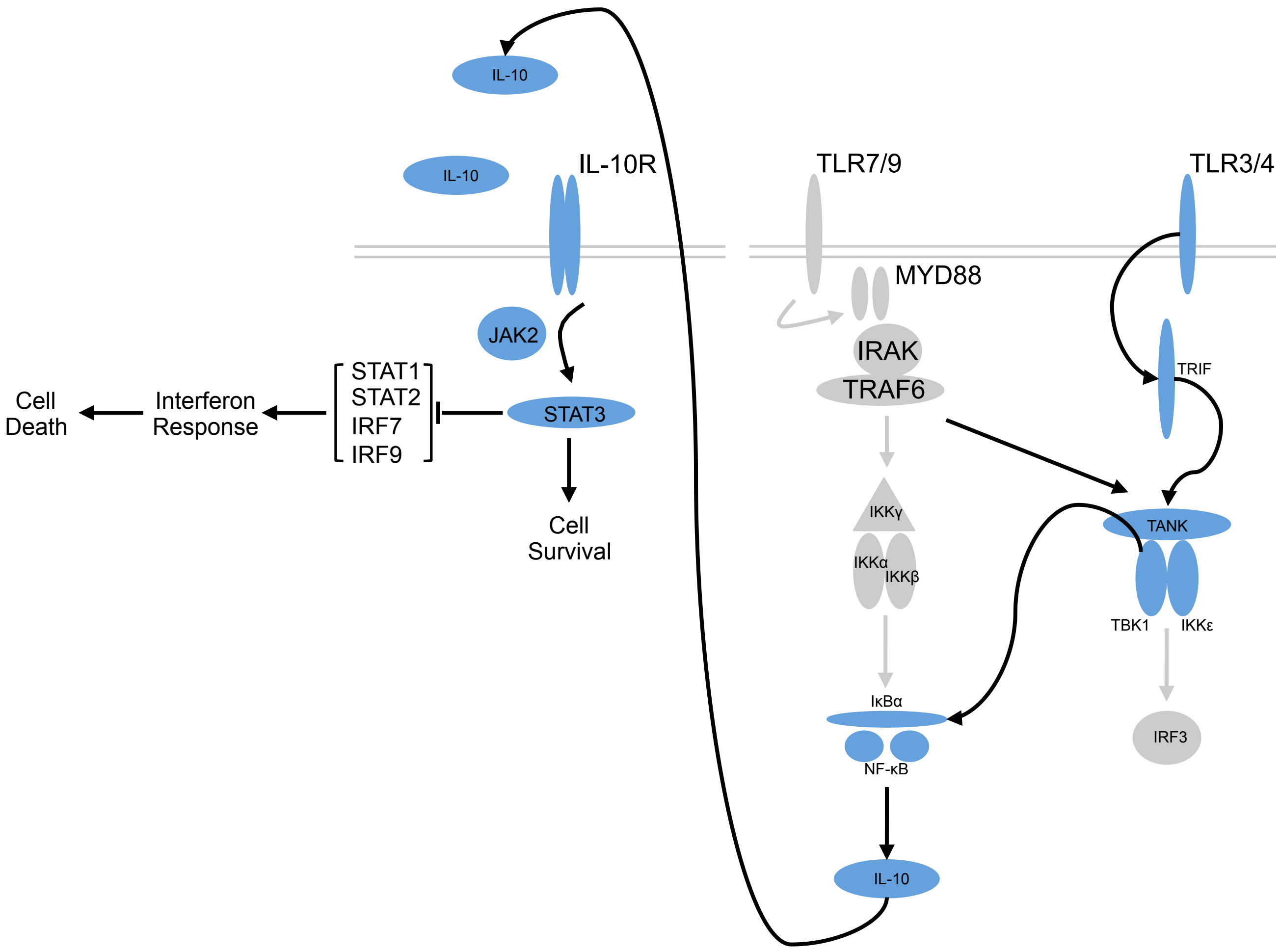

Supplementary Figure 2, Additional File 1

IKBKE and *TBK1* mRNA levels and overall survival of a cohort of patients with DLBCL. Using a publicly available dataset (Lenz et al., 2008) overall survival was analyzed by Kaplan-Meier curves for ABC-DLBCL and GC-DLBCL for patients expressing high and low levels (> median or < median) of *IKBKE* or *TBK1*. (A) TBK1 levels and ABC-DLBCL. (B) IKBKE levels and ABC DLBCL. (C) TBK1 levels and GC DLBCL. (D) IKBKE levels and GC DLBCL.

Figure S2

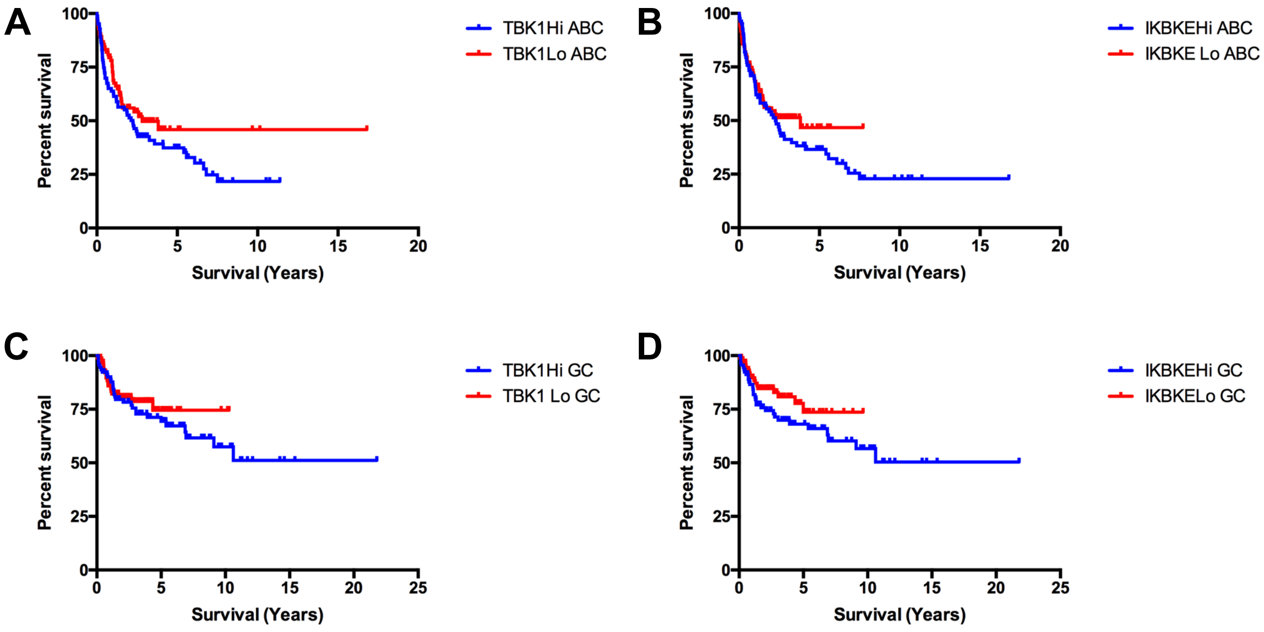

Supplementary Figure 3, Additional File 1
Cytokine production from Ly03 and Ly10 over time. **(A)** Multiplex ELISA (Luminex) to show levels of growth factors and chemokines at 8, 24 and 48 hours of culture of Ly03. **(B)** Multiplex ELISA (Luminex) to show levels of growth factors and chemokines at 8, 24 and 48 hours of culture of Ly10. n=1. Mean of duplicate samples.

Figure S3

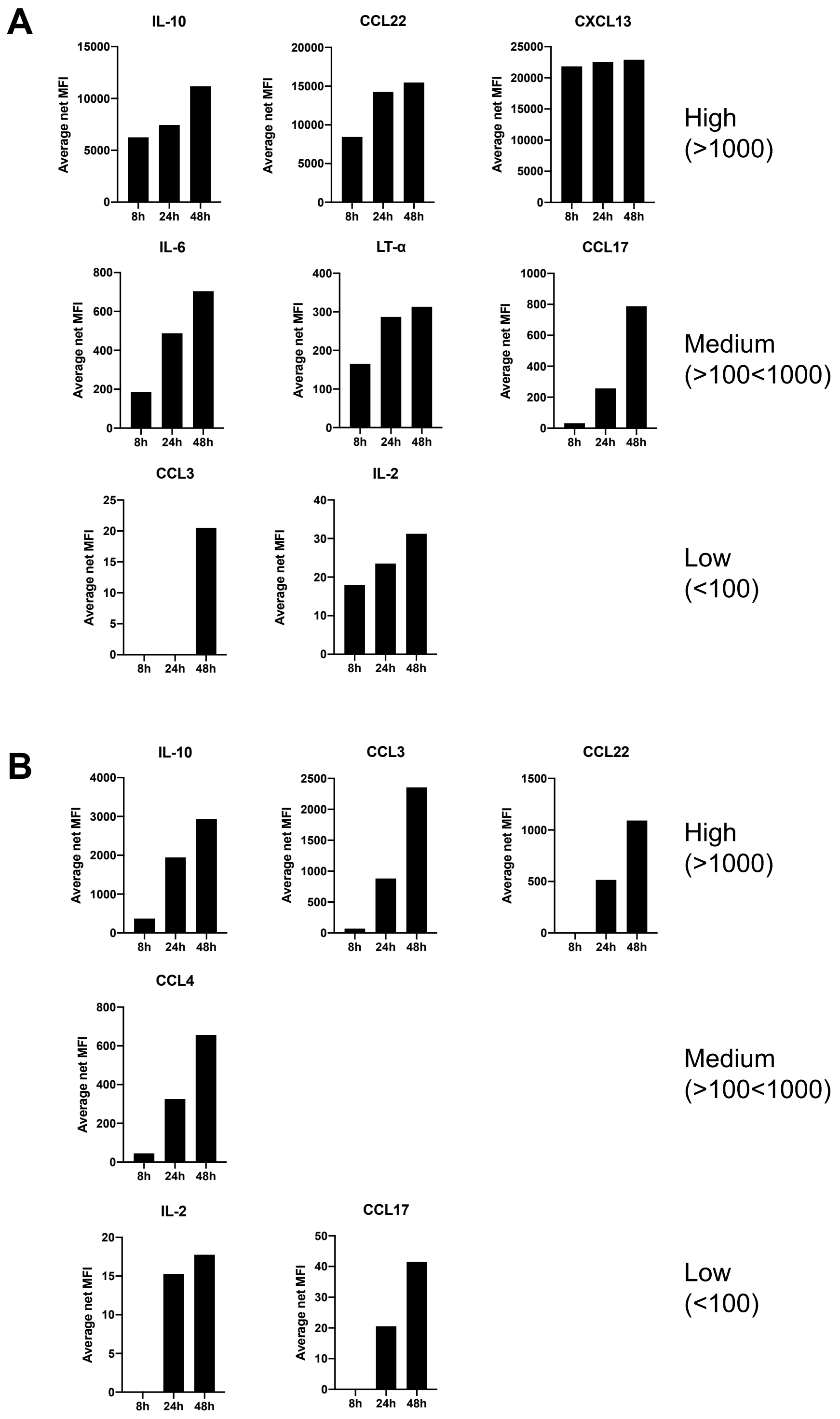

Supplement: Supplementary file 1 — Appendix S1 [file JCMM-24-11573-s001.pdf]
