# Supplementary material for: IKKε and TBK1 in diffuse large B‐cell lymphoma: A possible mechanism of action of an IKKε/TBK1 inhibitor to repress NF‐κB and IL‐10 signalling
Source: J Cell Mol Med. 2020 Aug 28;24(19):11573–82. doi: 10.1111/jcmm.15774 (PMC7576278; doi:10.1111/jcmm.15774)
Supplement: Supplementary file 2 — Appendix S2 [file JCMM-24-11573-s002.pdf]

Additional File 2

Carr M, Chapman K, Perrior T and Wagner SD

Figure 1E

Lane key:

1. Ly10
2. Ly03
3. Pfeiffer
4. Toledo
5. Ly19
6. SUDHL4
7. SUDHL6

1

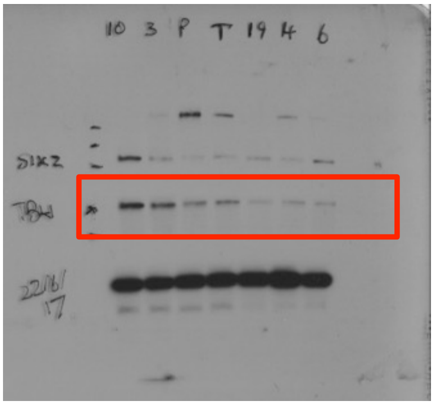

TBK1

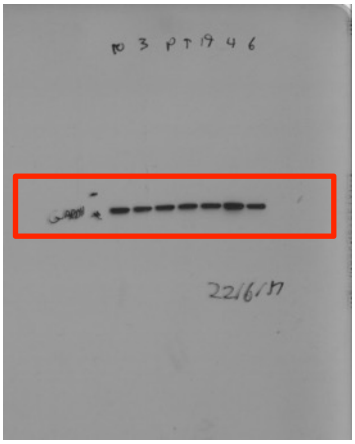

GAPDH

Lane key:

1. Ly10
2. Ly03
3. Pfeiffer
4. Toledo
5. Ly19
6. SUDHL4
7. SUDHL6

2

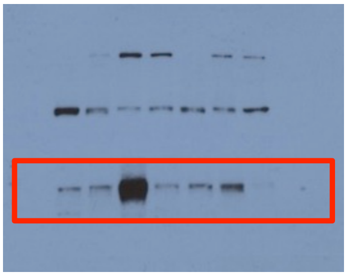

IKKε

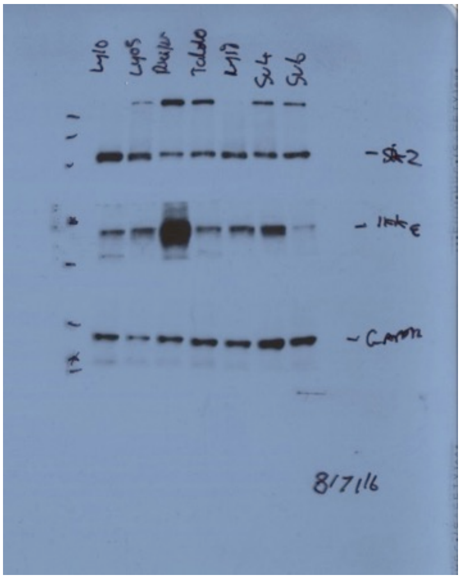

GAPDH

Figure 1F

Lane key:
1. Ly10
2. Ly03
3. Pfeiffer
4. Toledo
5. Ly19
6. SUDHL4
7. SUDHL6

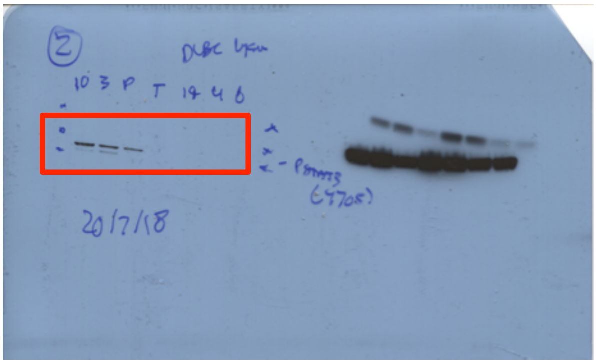

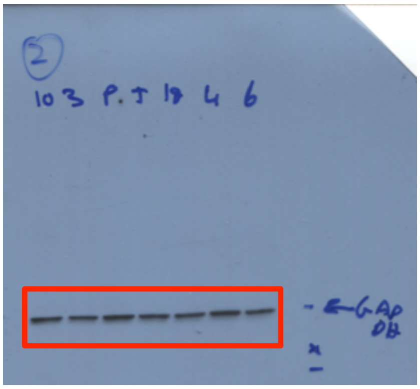

Lane key:
1. Ly10
2. Ly03
3. Pfeiffer
4. Toledo
5. Ly19
6. SUDHL4
7. SUDHL6

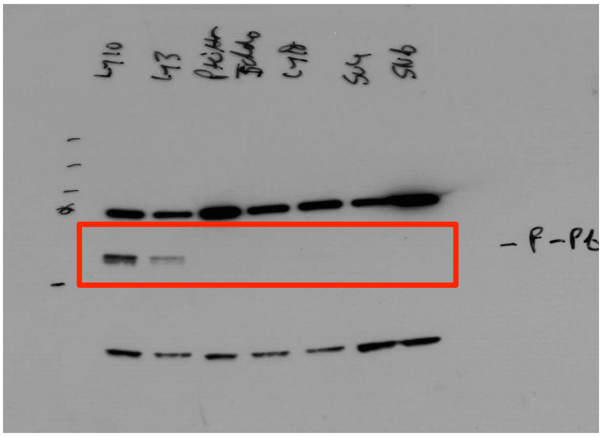

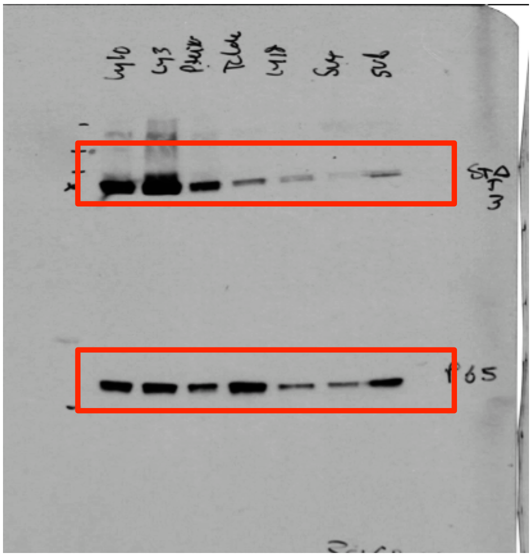

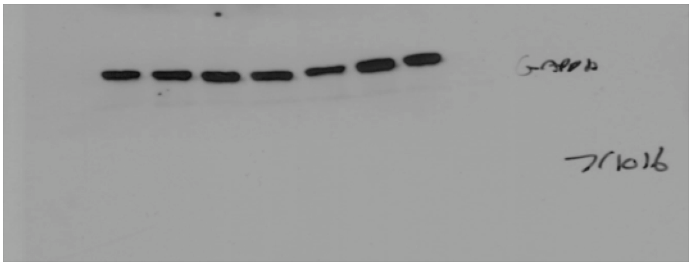

Figure 2F

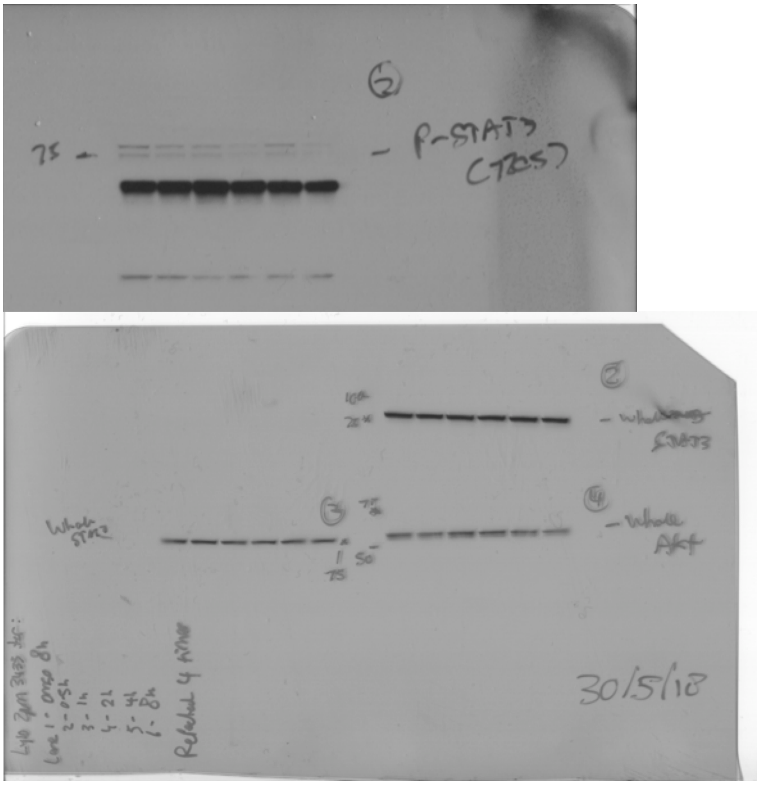

Lane key:

1. Ly10 + DMSO
2. Ly10 + DMX3 2µM (0.5h)
3. Ly10 + DMX3 2µM (1h)
4. Ly10 + DMX3 2µM (2h)
5. Ly10 + DMX3 2µM (4h)
6. Ly10 + DMX3 2µM (8h)

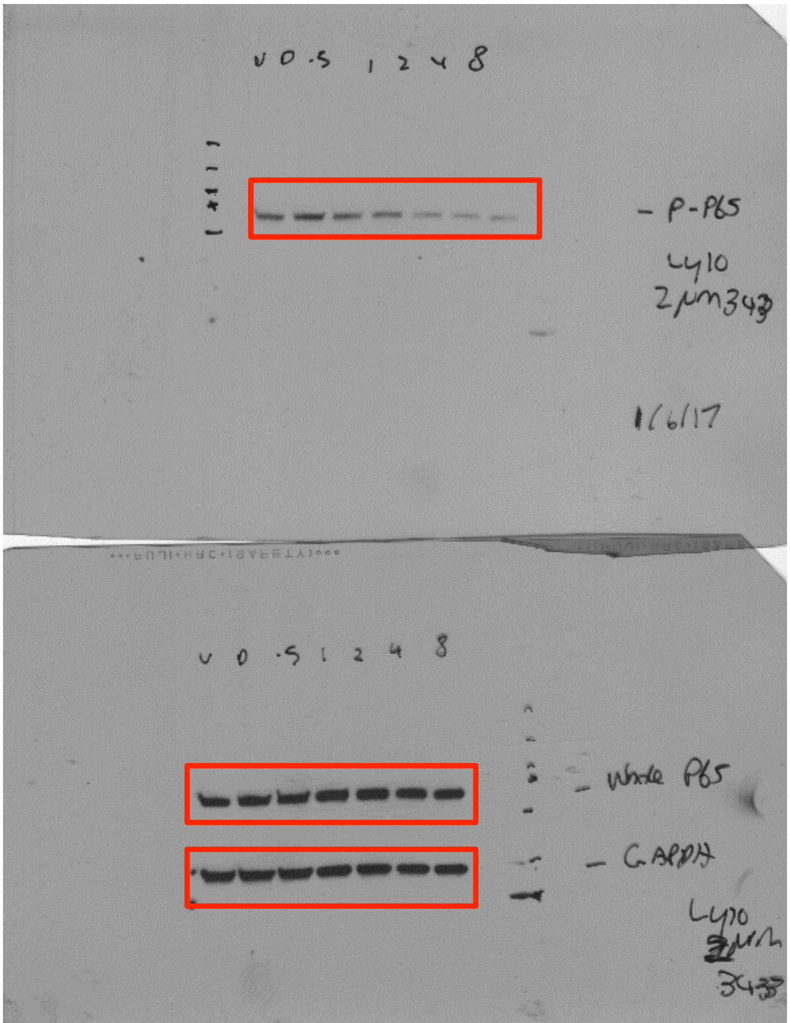

Lane key:

1. Ly10 + DMSO
2. Ly10 + DMX3 2µM (0.5h)
3. Ly10 + DMX3 2µM (1h)
4. Ly10 + DMX3 2µM (2h)
5. Ly10 + DMX3 2µM (4h)
6. Ly10 + DMX3 2µM (8h)

Figure 2G

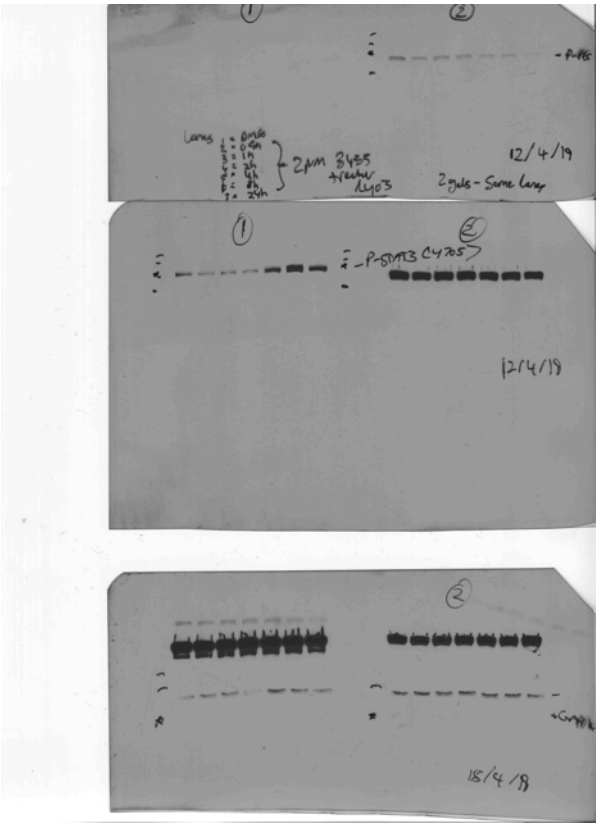

Lane key:

1. Ly03 + DMSO
2. Ly03 + DMX3 2µM (0.5h)
3. Ly03 + DMX3 2µM (1h)
4. Ly03 + DMX3 2µM (2h)
5. Ly03 + DMX3 2µM (4h)
6. Ly03 + DMX3 2µM (8h)
7. Ly03 + DMX3 2µM (24h)

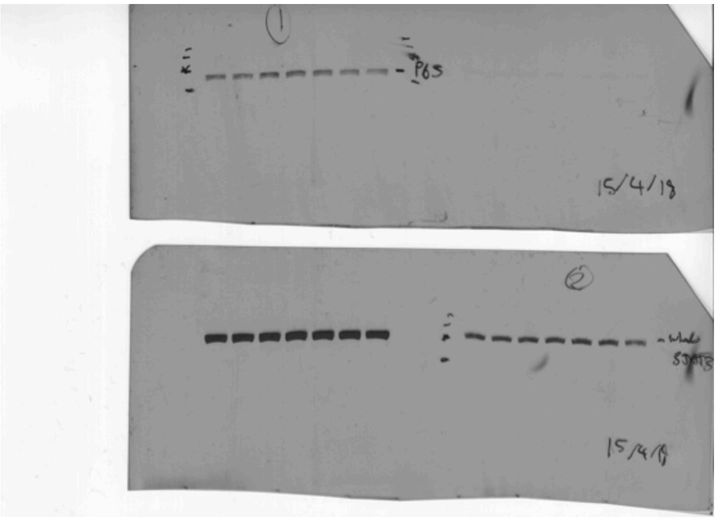

Figure 3C

Lane key:

1. Ly10 + DMSO+PBS
2. Ly10 + DMSO + CCL3 100ng/ml
3. Ly10 + DMSO + CCL4 100ng/ml
4. Ly10 + DMSO + IL10 50ng/ml
5. Ly10 + DMX3 2µM (8h) + PBS
6. Ly10 + DMX3 2µM (8h) + CCL3 100ng/ml
7. Ly10 + DMX3 2µM (8h) + CCL4 100ng/ml
8. Ly10 + DMX3 2µM (8h) + IL10 50ng/ml

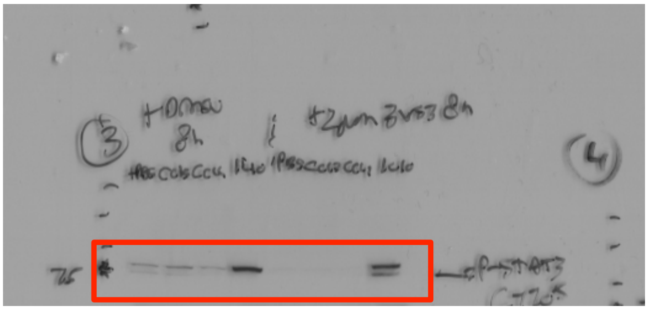

pSTAT3 (Y705)

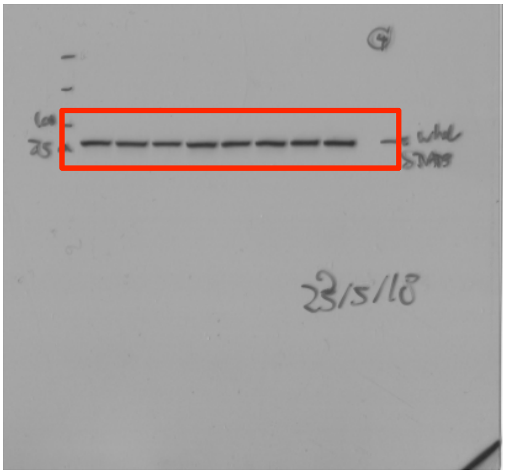

STAT3

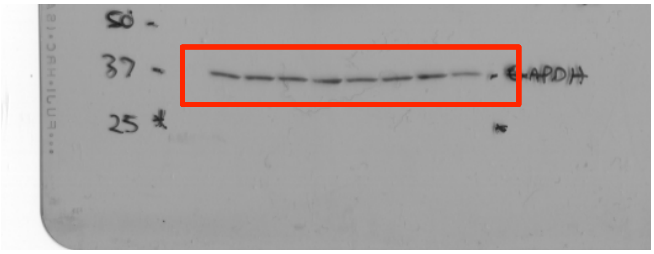

GAPDH

Figure 4B

Lane key:

1. PDX 0257
2. PDX 2214
3. PDX 2345
4. PDX 2318

TBK1

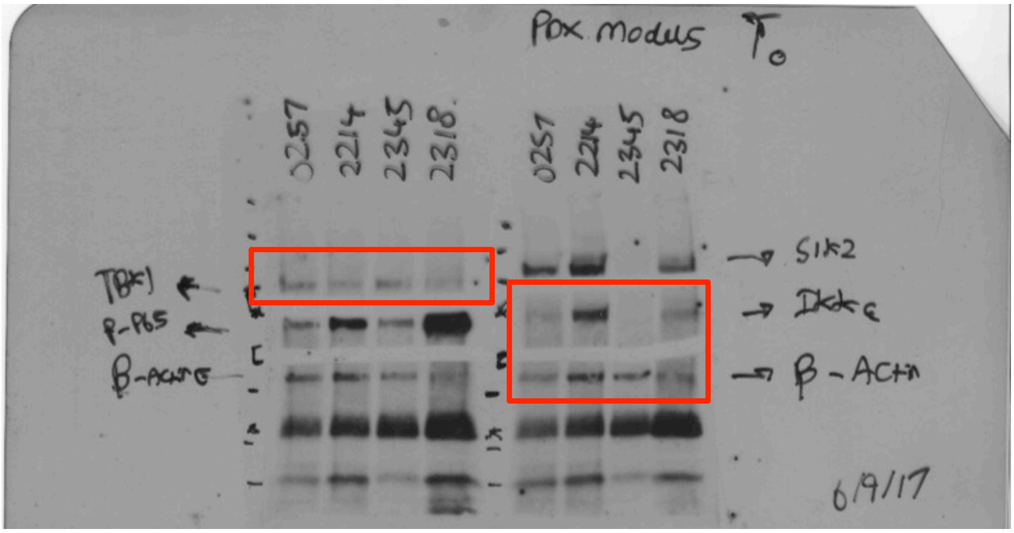

IKKε

B-Actin

P-STAT3

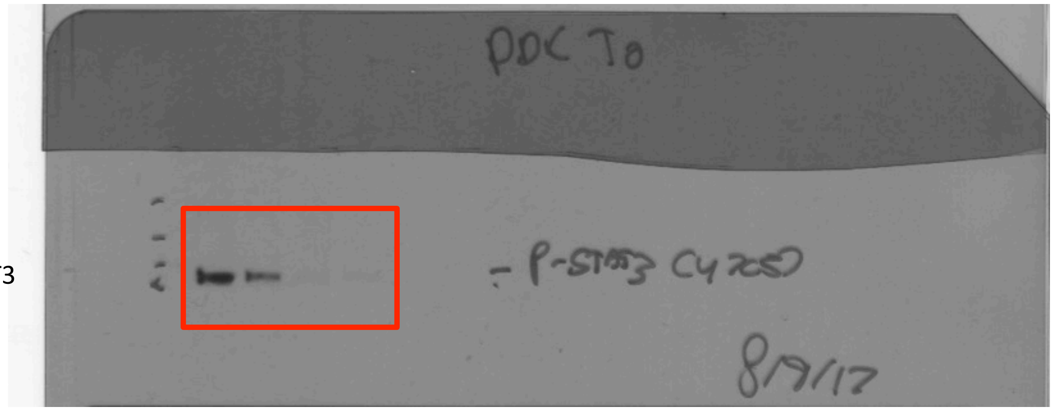

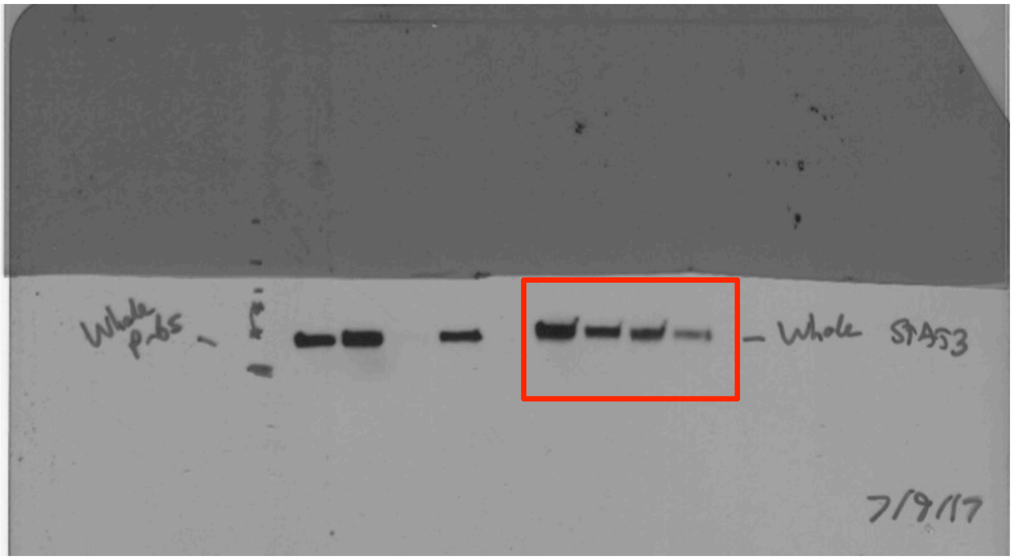

STAT3
